# Influenza and pneumococcal vaccine hesitancy in the elderly population: results from two representative surveys in Germany

**DOI:** 10.1186/s12889-025-22441-9

**Published:** 2025-05-06

**Authors:** Dorothee Heinemeier, Philipp Schmid, Sarah Eitze, Cornelia Betsch, Vaccination60+ study group

**Affiliations:** 1 Institute for Planetary Health Behaviour, University of Erfurt, Erfurt, 99089 Germany; 2 Implementation Research, Health Communication, Bernhard-Nocht-Institute for Tropical Medicine, Hamburg, Germany; 3 Centre for Language Studies, Radboud University, Nijmegen, The Netherlands

**Keywords:** Vaccination, Behavioral insights, Vaccine behavior, Vaccine

## Abstract

**Background:**

The reasons for low influenza and pneumococcal vaccine acceptance in the elderly population are largely unknown – despite the great need of vaccines in this risk group. While many studies examine the relationship between factors influencing vaccination, such as sociodemographic characteristics and influenza and pneumococcal vaccination intentions and behavior, psychological factors, such as vaccine-specific attitudes, are underutilized in research on vaccination behaviors and intervention strategies. This article assesses the psychological antecedents of influenza and pneumococcal vaccination in the elderly and assesses the predictive power of psychological vs. sociodemographic and other factors surrounding vaccination, on vaccination behavior.

**Methods:**

A cross-sectional telephone survey, representative of age, gender and rural/urban residence, was conducted with* N* = 701 German participants > 60 years of age, during the influenza season of 2016–17. Multiple logistic regressions were conducted to identify the relevant determinants of vaccination behavior.

**Results:**

Results show unique patterns in the psychological antecedents: while confidence, the belief in the effectiveness of vaccination and calculation, the need for information, complacency, the lack of risk perception and constraints, and perceived practical barriers to vaccination predicted influenza vaccination behavior, only complacency predicted pneumococcal vaccination behavior. The amount of explained variance in influenza vaccination behavior nearly doubles when psychological antecedents of vaccination are taken into account, beyond other factors surrounding vaccination. However, the effect was smaller for pneumococcal vaccination behavior. The results are compared to a subnational sample.

**Conclusions:**

Understanding the psychological drivers of vaccination can help to plan interventions effectively.

**Trial registration:**

Deutsches Register Klinische Studien (German Clinical Trials Register) DRKS00012653. Registered 24.11.2017. Retrospectively registered.

**Supplementary Information:**

The online version contains supplementary material available at 10.1186/s12889-025-22441-9.

The pandemic has shown how critical individual behavior is to the success of public health interventions. In 2022, the WHO Euro member states agreed on a resolution on behavioral and cultural insights to help countries monitor people's perceptions and social and cultural circumstances [1]. This shall improve the countries’ public health decisions and ensure equitable access to health. Moreover, the COVID- 19 pandemic might have affected how people think and feel about public health measures, such as vaccination. Already before the pandemic, vaccine uptake was low for some vaccines in some risk groups. It is important to understand the challenges that existed already before the pandemic to serve as a benchmark for future research, understand changes and improve intervention design. This work therefore reports data from before the pandemic and focuses on people over 60 years of age regarding their perceptions of influenza and pneumococcal vaccination.

Influenza and pneumococcal disease are significant causes of morbidity and mortality around the globe [2, 3]. Chronically ill patients, young children and the elderly population are at specific risk for severe complication following pneumococcal and influenza infections [4–7]. Before the influenza activity decreased due to COVID- 19 pandemic-related mitigation measures, it was estimated that there are 3,000,000–5,000,000 severe influenza cases and 290,000–650,000 deaths due to influenza worldwide each year [8, 9]. Most of the influenza-associated deaths were among the elderly population above 60 years of age [10]. Influenza and pneumococcal vaccines reduce the risk of hospital admission and in-hospital mortality, and receiving both vaccines has an additive effect on risk reduction [10, 11]. In many countries, influenza and pneumococcal vaccines are recommended for individuals aged 60 years or older [10].

However, despite the availability of safe vaccines, vaccine uptake rates of influenza are still far below the recommended target of 75% in all at risk groups in all EU countries [10, 12]. Influenza uptake rates among older adults range between 10% in Poland and 75% in the US, in 2021 [13, 14]. The uptake rates for pneumococcal vaccines in the age group range between 20% in Australia for individuals aged 71–79 and 70% in UK for adults older than 65, in 2021 [15, 16]. For Germany, rates were especially low for pneumococcal vaccination, around 17% in 60–67 years old’s in 2022 [17]. For the 2015–2019 seasons, this rate was in the 10% range, and increased thereafter [17]. The influenza vaccination uptake rate was at 43% for the 2021–2022 season. This rate has been largely the same in the seasons before, from 2014–2015 and 2019–2020 seasons, when it was 39% [18, 19].

This “delay in acceptance or refusal of vaccination despite availability of vaccination services” has been described as vaccine hesitancy [20]. “Vaccine hesitancy is complex and context specific, varying across time, place and vaccines. It is influenced by factors such as complacency, convenience and confidence” [20]. In order to make vaccine hesitancy measurable, Betsch and colleagues linked these factors to psychological health behavior theories and expanded the list of relevant factors by developing a scale – the 5 C psychological antecedents of vaccination [21]. The antecedents are: confidence, constraints, calculation, collective responsibility and complacency. The antecedents serve as broad categories relevant for vaccination behavior, further described as macro-level factors, that capture and describe theoretically relevant psychological constructs on a more fine-grained level, further described as micro-level factors.

*Confidence* “is defined as trust in (i) the effectiveness and safety of vaccines, (ii) the system that delivers them, including the reliability and competence of the health services and health professionals, and (iii) the motivations of policy-makers who decide on the need of vaccines” [20]. On a micro-level, individuals who lack confidence in vaccination have negative attitudes towards vaccination and low knowledge about vaccination [22]. *Constraints* is defined as issues with “physical availability, affordability and willingness to pay, geographical accessibility (…)” [20]. These individuals can have positive attitudes towards vaccination in general but perceive their behavioral control as too low to take action [22]. *Calculation* describes a weighing process of risk and benefits of getting vaccinated. These individuals "engage in an extensive information search for pros and cons of vaccination" [22] that can explain behavior like fence sitting i.e. hesitancy due to the consumption of an equal amount of pro and anti-vaccination information. This type has no strong negative attitude towards vaccination per se but rather refuses vaccination based on the perceived utility. *Collective responsibility* describes the situation whereby people understand the value of, and engage in vaccination, to contribute to herd immunity. This antecedent is related to empathy and communal orientation and captures the pro-social willingness to also protect unvaccinated individuals in society by getting vaccinated. *Complacency* “exists where perceived risks of vaccine preventable diseases are low and vaccination is not deemed a necessary preventive action” [20]. The perceived risk of the disease, awareness for and knowledge about the disease are generally low. Moreover, individuals who are complacent do not have a strong attitude towards the vaccine and do not perceive vaccination as a social norm [22].

At the time of the study, the reasons for low uptake rates and vaccine hesitancy for seasonal influenza and pneumococcal vaccination among the elderly population in Germany were largely unknown. This makes it difficult to design and target interventions to positively influence changes in vaccination behavior. For example, a systematic review found that most studies focus on sociodemographic variables to understand the reasons for low vaccine uptake and lack a connection to psychological health behavior theories that provide essential reasons of low vaccine acceptance – and levers to overcome it [23]. In the case of pneumococcal disease only very few studies exist that address barriers of vaccine uptake at all. The existing research suggests that knowledge and awareness about the vaccine are major barriers of pneumococcal vaccine uptake in Germany [24]. However, the study reveals little about which factors need to be addressed in a potential intervention.

The goal of this study is to understand and measure the relationship between 5 C psychological antecedents of influenza and pneumococcal vaccination and vaccination intention and behavior in the elderly population and analyze the predictive power of the antecedents compared to other factors influencing vaccination, e.g. sociodemographic and contextual variables.

## Methods

A cross-sectional representative survey with the elderly population in Germany (referred to as national sample) was conducted during the 2016–2017 influenza season with a questionnaire that covered awareness and influenza knowledge, the antecedents of vaccination, vaccination intention and behavior. A second survey with the purpose of developing a health campaign to increase vaccination uptake was conducted in the federal state of Thuringia (referred to as subnational sample) using the same methods. The reasoning behind selecting the study sample is detailed in the study protocol [25]. The research reported in this article received Institutional Review Board approval by the University of Erfurt (No 17/05/29). Verbal informed consent was obtained by the interviewer. Participation could be abandoned every time. The authors had no access to information that could identify individual participants during or after data collection.

### Participants

Our sample consisted of *N* = 701 participants in the national and *N* = 700 in the subnational sample. The sample size was based on an a-priori power analysis (independent t-test: power 0.8, alpha 0.05, effect size d = 0.2) and rounded to the nearest higher hundred. Further details and evidence that informed this decision are mentioned in the corresponding study protocol [25]. Representativeness of the sample was established using gender, age, education and residence in an urban or rural area as quota variables (Table 1).

**Table 1:** Sample characteristics (unweighted and weighted data)

	**National Sample N (%)**		**Subnational Sample N (%)**	
**Characteristics**	**Unweighted**	**Weighted**	**Unweighted**	**Weighted**
Age				
60–64	155 (22.1)	162 (23.1)	153 (21.9)	173 (24.7)
65–74	288 (41.1)	258 (36.8)	262 (37.4)	261 (37.3)
75 +	258 (36.8)	281 (40.1)	285 (40.7)	266 (38.0)
Gender				
male	318 (45.4)	313 (44.7)	253 (36.1)	308 (44.0)
female	383 (54.6)	388 (55.3)	447 (63.9)	392 (56.0)
Educational qualification				
low	372 (53)	590 (85)	335 (47.9)	587 (83.9)
medium	146 (20.8)	46 (6.6)	123 (17.6)	32 (4.5)
high	167 (23.8)	50 (7.2)	222 (31.7)	63 (9.0)
no data	16 (2.2)	14 (2)	20 (2.9)	18 (2.6)
Influenza vaccination	341 (48.6)	332 (47.4)	352 (50.3)	365 (52.1)
Pneumococcal vaccination	139 (19.8)	141 (20.1)	190 (27.1)	168 (24.0)

We coded educational qualification as low, medium, high using the International Standard Classification of Education 97 (ISCED- 97) [26]. Data was weighted according to [27]

### Procedure

**Fig. 1:**
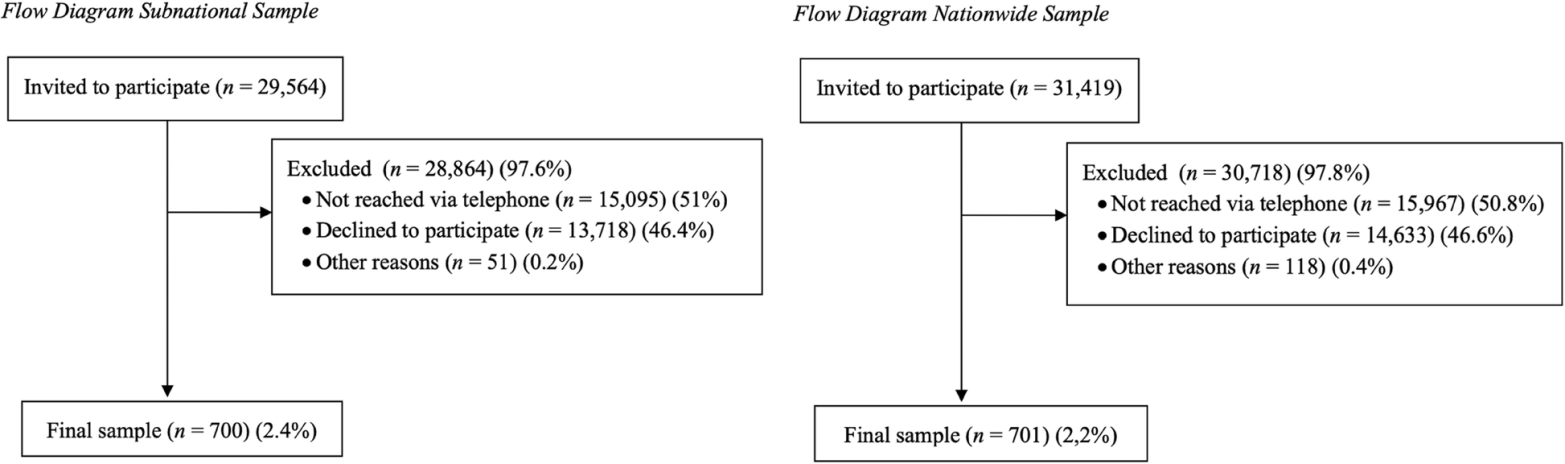
Flow diagram of participants

Between October 2016 and December 2016, households were contacted by a professional survey company (Institute for Applied Marketing and Communication Research, IMK) using random digital dialing. Figure 1 shows the participants selection process for both samples. Inclusion criteria were participants age (≥ 60 y/o) and being fluent in the German language. If this was the case, contacted individuals were asked to participate in the survey. Those who agreed, were interviewed, using a computer-assisted telephone interview software (CATI).

### Measures

All measures used in the surveys are presented in Table 2. The questionnaire also contained questions about media use and sepsis knowledge (see results here: [28, 29]). Data on participants’ awareness about influenza, pneumococci and respective vaccinations, knowledge about influenza and influenza vaccination and current and previous vaccination intention and behavior (self-report) was collected. Influenza vaccination intention was only assessed in participants who indicated not having been vaccinated in the season (yet). We assessed the 5 C psychological antecedents of influenza and pneumococcal vaccination as well as sociodemographic and other factors surrounding vaccination [21]. All of the factors were chosen based on previous research that showed their meaningful relationship on vaccination behavior [23]. We dummy coded educational qualification (low = 1, medium = 2, high = 3), marital status (married = 1, single = 2, widowed = 3), the frequency of doctor visits (less than 2–3 months = 1, every 2–3 months = 2, more than every 2–3 months = 3) and duration to reach the doctor (less than 5 min = 1, 6–10 min = 2, longer than 10 min = 3). The questionnaire was developed after reviewing the literature and conducting informal interviews with medical experts. The questionnaire was pre-tested for clarity and length with *n* = 30 participants.

**Table 2:** Measures used in the survey

Construct	Sample Item and Source	Answer Format	Number of Items
Awareness of influenza [pneumococci] (vaccination)	Have you ever heard about influenza [pneumococci] (vaccination)?	Yes/no/don’t know	4 items
Influenza vaccination behavior	‘Did you get vaccinated against influenza this season [fall 2016]l?’ Adapted from [30]	Yes/no	2 items
Pneumococcal vaccination behavior	‘Did you get vaccinated against pneumococcal infection in the past 10 years?’	Yes/no/don’t know	1 item
Influenza vaccination intention	‘Do you intent to get vaccinated against influenza this season? ‘ Adapted from [30]	5-point Likert-scale (definitely not vaccinate/definitely vaccinate)	2 items
5 C psychological antecedents of influenza [pneumococcal] vaccination (short scale)	Confidence: ‘I am completely confident that the influenza [pneumococcal] vaccine is safe’ [21]	5-point Likert-scale (strongly disagree/strongly agree)	1 item per antecedent (10 items)
Knowledge about influenza	‘The efficacy of influenza vaccinations has been proven.’ Adapted from [31, 32]	Yes/no/don’t know	9 items integrated in Influenza knowledge sum score
Recommendation from doctor	Did your doctor recommend the influenza [pneumococcal] vaccination to you?	Yes/no	1 item each
Frequency of interaction with health service	How often do you go to your doctor, per year?	6-point Likert-scale (at least once a week/several times in a month/once a month/once every 2–3 months/once every 4–6 months/rarely or never)	1 item
Duration to reach doctor	How long does it normally take you need to get to your doctor?”)	7-point Likert-scale (less than 5 min/6–10 min/11–20 min/21–30 min/31–40 min/41–50 min/51–60/longer than an hour)	1 item
Perceived health status	How would you describe your health status in general?	5-point Likert-scale (excellent/bad)	1 item
Chronic disease	Do you have a chronic illness?	Yes/no	1 item
Age	How old are you?	Open answer	1 item
Gender	What is your gender?	male/female	1 item
Educational qualification	What is your highest educational qualification? (ISCED 97 classification) [26]	Low/medium/high/no data; low = no educational qualification; secondary school, medium = A-levels, high = university degree (Bachelor//Master/Diplom)	1 item
Job status	Are you currently employed?	Yes/no	1 item
Family status	Are you currently…?	Married/single/widowed	1 item
Health Insurance	What is the status of your health insurance?	Statutory/private health insurance	1 item
Town size	What is the size of your town?	Small = below 10.000, large = above 10.000 inhabitants	1 item
Children	Do you have children?	Yes/no	1 item
Living conditions	Do you live together with your partner(/with your children?	Yes/no	1 item

### Statistical analyses

The statistical analyses were conducted using SPSS version 25. We used weighted data to present the distribution of participants’ sociodemographic factors (Table 1), awareness and influenza knowledge (Table 3). Data was weighted to match the census data according to the criteria age, gender, educational level, and urban/rural residency according to [27]. We used unweighted data for all procedures that required statistical inference [33]. Multiple regressions were conducted to identify relevant correlates of previous influenza and pneumococcal vaccination behavior (logistic regression) and determinants predicting the influenza vaccination intention (linear regression) (step 1: sociodemographic, contextual and physical variables, step 2: 5 C psychological antecedents of vaccination).

**Table 3:** Awareness and influenza knowledge (weighted data)

	**National Sample N (%)**			**Subnational Sample N (%)**		
**Items**	**Yes**	**No**	**Unsure**	**Yes**	**No**	**Unsure**
Awareness						
Have you ever heard about influenza?	701 (100)	-	-	700 (100)	-	-
Is there a vaccination against influenza?	688 (98.2)	8 (1.1)	5 (0.7)	684 (97.7)	15 (2.1)	1 (0.2)
Have you ever heard about pneumococcal disease?	553 (78.9)	140 (19.9)	8 (1.1)	603 (86.1)	93 (13.3)	4 (0.5)
Is there a vaccination against pneumococcal disease?^a^	256 (36.5)	125 (17.8)	172 (24.6)	334 (47.8)	95 (13.6)	173 (24.7)
Items of the knowledge score	*M* = 0.632, *SD* = 0.212			*M* =.628, *SD* =.189		
The effectiveness of the influenza vaccination varies from year to year	454 (64.8)	104 (14.8)	138 (19.7)	453 (64.8)	97 (13.8)	149 (21.2)
An influenza shot can [not] get me influenza	297 (42.4)	334 (47.6)	68 (9.8)	313 (44.7)	309 (44.1)	77 (11.0)
To be protected against influenza, you have to get vaccinated each year	610 (87)	63 (8.9)	23 (3.3)	623 (89.0)	49 (7.0)	27 (3.8)
Influenza is [not] a severe cold	279 (39.8)	392 (56)	28 (4.1)	389 (55.6)	294 (42.0)	17 (2.5)
Additives in the influenza vaccination are [not] dangerous	148 (21.1)	249 (35.5)	297 (42.4)	139 (19.8)	265 (37.9)	295 (42.2)
Influenza vaccinations [do not] promote allergies	81 (11.5)	334 (47.6)	281 (40.1)	66 (9.5)	381 (54.5)	247 (35.3)
Influenza vaccinations are [not] unnecessary, since influenza can be treated well	68 (9.6)	574 (82)	54 (7.6)	52 (7.5)	593 (84.7)	54 (7.7)
The efficacy of the influenza vaccination has been proven	522 (74.5)	69 (9.8)	107 (15.3)	542 (77.4)	34 (4.9)	121 (17.3)
Influenza can cause pneumonia	499 (71.2)	83 (11.9)	115 (16.4)	495 (70.7)	117 (16.7)	87 (12.5)

^a^This question filtered participants who heard about pneumococcal disease before

We conducted a missing data analysis to evaluate the potential impact of missing values on our results (see Supplement). Supplement 1 presents the missing data analysis, including an assessment of whether individuals included in the analysis differ significantly from those excluded based on any of the analysis variables (Tables S1–S6). Supplement 2 details the imputation methods used to address missing data (Tables S7–S13). To address the issue of missing data, we imputed missing values to ensure the dataset remained suitable for analysis without discarding valuable information. For continuous or interval-scale variables, we replaced missing values with the mean of the observed values for that variable (mean imputation). For categorical and binary (dummy) variables, we replaced missing values with the most frequently occurring category within that variable (mode imputation).

## Results

The data set and syntax of all of the following analyses are available in the Open Science Framework repository (private, view-only link) https://osf.io/8my5k/.

### Awareness and knowledge

**Fig. 2:**
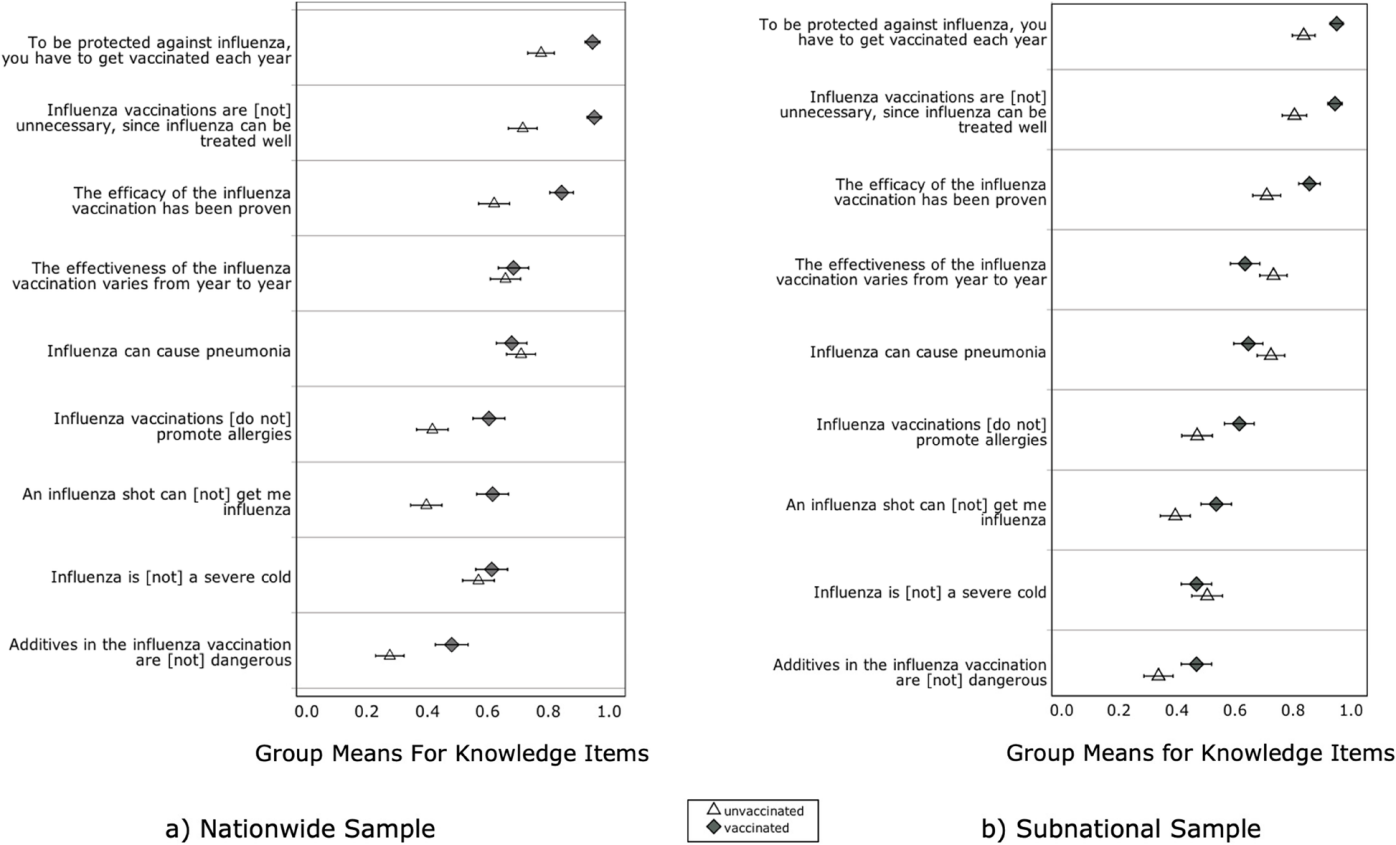
Mean influenza knowledge. *Note.* The figure presents the group means for knowledge items (0 = incorrect, 1 = correct) in the (**a**) national sample and (**b**) subnational sample, separated for participants’ vaccination behavior (unvaccinated [triangle] vs. vaccinated [diamonds]). Error bars show 95% CIs

We assessed awareness and influenza knowledge to understand and identify potential gaps and misconceptions about influenza (Table 3 and Fig. 2). While most of the participants had heard of influenza, pneumococcal disease and influenza vaccination before, only a small proportion of participants had heard of pneumococcal vaccination. For assessing influenza knowledge, we calculated a mean score that integrated the knowledge items (0 = incorrect to 1 = correct). Overall, participants answered more than half of the knowledge items correctly.

### Vaccination behavior

In the national sample, 48.6% (*n* = 341) of participants reported being vaccinated against influenza and 19.8% (*n* = 139) against pneumococci in the last 10 years. In the subnational sample, 50.3% (*n* = 352) of participants reported being vaccinated against influenza and 27.1% (*n* = 190) against pneumococci.

#### 5C psychological antecedents of vaccination as determinants of vaccination behavior and intention

Influenza vaccination intention was only assessed when participants indicated that they had not yet received vaccination in the current influenza season. For the analysis of previous pneumococcal vaccination behavior, we only included participants who heard about pneumococcal disease before. Multiple logistic and linear regressions were conducted to predict influenza and pneumococcal vaccination behavior and influenza vaccination intention based on the psychological antecedents of vaccination and other relevant factors that have been shown to affect vaccination behavior previously. We used a stepwise approach with sociodemographic and other factors surrounding vaccination in step 1 and the 5 C psychological antecedents of vaccination in step 2. Variables included age, physical health (higher values indicate higher age, better health); education (low (ref), medium, high), marital status (married (ref), single, widowed), frequency of doctor visit (less than every 2–3 months (ref), every 2–3 months, more than every 2–3 months), duration to reach doctor (less than 5 min (ref), 6–10 min, longer than 10 min); dichotomous variables: gender (male vs. female), job status (retired vs. employed), insurance (statutory vs. private), town size (small, large), living with partner (yes vs. no), having children (yes vs. no), being chronically ill (no vs. yes), recommendation from doctor (no vs. yes). The 5 C psychological antecedents complacency and collective responsibility were recoded. Higher values for complacency indicate that participants are more complacent and feel less at risk to influenza. Higher values for collective responsibility indicate higher prosocial motivation to get vaccinated.

The pattern of results from the missing data analysis shows that socio-demographic factors fluctuate to some extent, while psychological determinants remain relatively stable (Supplement, Tables S8-S13). The 5 C framework is largely unaffected by data imputation, with results remaining consistent for behavior and intention. However, an exception is observed in the 5 C calculation in the analysis of influenza uptake from the subnational sample, where calculation was significant in the original, but not in the imputed data analysis.

##### National sample

**Fig. 3:**
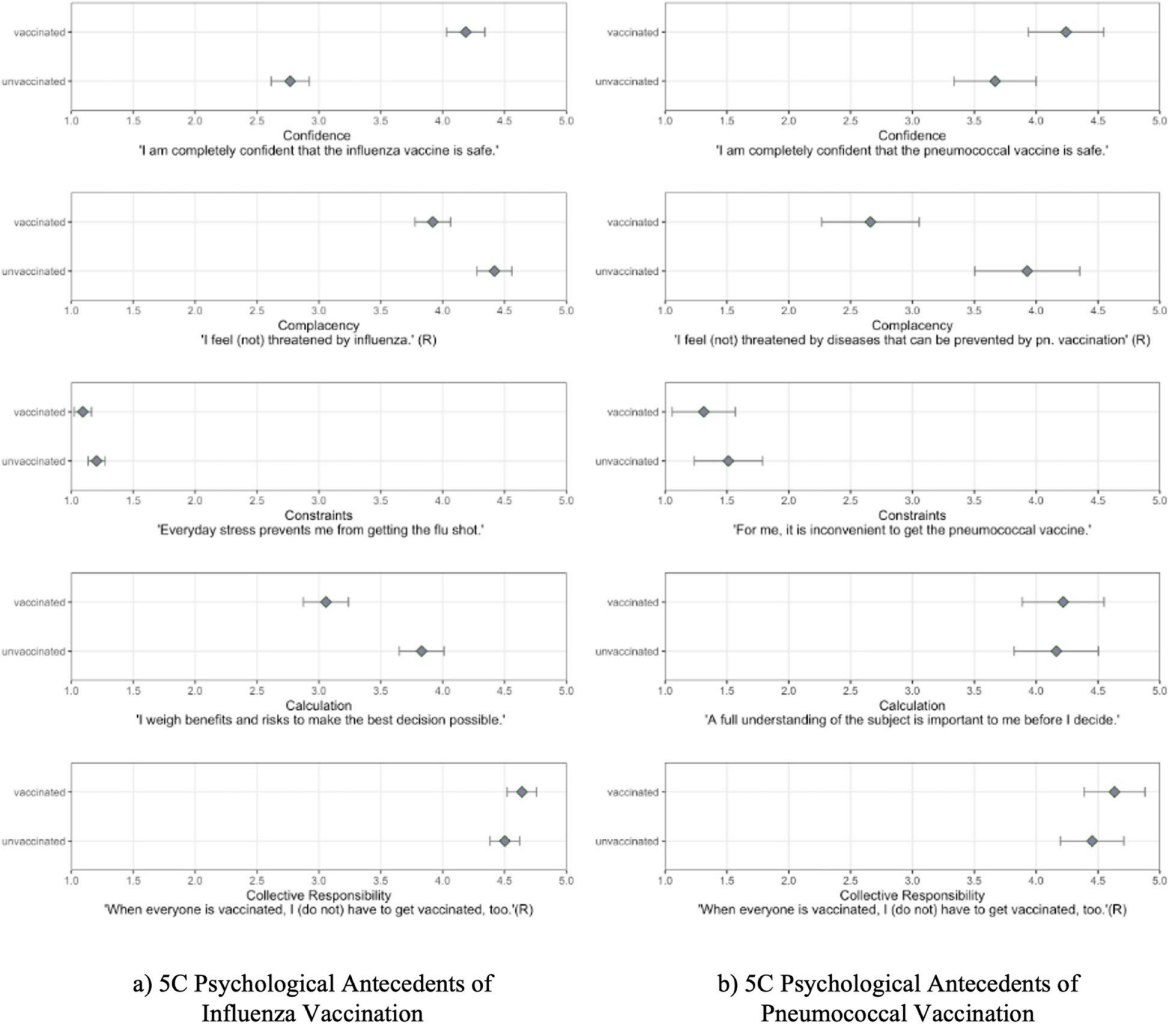
5 C psychological antecedents of vaccination in the national sample. *Note.* The Figure shows estimated mean differences and 95% CIs for the 5 C psychological antecedents of vaccination between unvaccinated vs. vaccinated participants. **a** Influenza vaccination. Participants vaccinated against influenza show higher confidence, are less complacent, have less constraints and engage less in the calculation of risks and benefits of influenza vaccination compared to unvaccinated participants. **b** Pneumococcal vaccination. Participants vaccinated against pneumococci are less complacent compared to unvaccinated individuals. Results are obtained from the analysis of regression in Table 4

Table 4 presents the results from the stepwise regressions; Fig. 3 shows the mean scores for all antecedents for vaccinated and unvaccinated participants. The stepwise approach where the 5 C psychological antecedents of vaccination were added after all other factors showed that including the 5 C accounted for an additional amount of variance in influenza ($\Delta$ *R*^2^ = 0.290) and pneumococcal ($\Delta$ *R*^2^ = 0.08) vaccination behavior beyond sociodemographic and other factors (Table 4). We will first report the results the 5 C antecedents for all outcomes, then for sociodemographic and the other factors.

**Table 4:** Results of regression analyses for the determinants of vaccination behavior and intention in the national sample

	**Influenza Vaccination Behavior**						**Influenza Vaccination Intention**							**Pneumococcal Vaccination Behavior**					
	**Step 1**			**Step 2**			**Step 1**			**Step 2**				**Step 1**			**Step 2**		
**Determinants**	**B**	**SE**	**OR**	**B**	**SE**	**OR**	**B**	**SE**	**β**	**B**		**SE**	**β**	**SE**	**OR**	**B**	**B**	**SE**	**OR**
(Constant)	− 3.321	1.496	.036	− 3.105	2.019	.045	.926	1.322		1.839		1.315		− 14.752	4.663	.000	− 16.273	5.722	.000
Age	.034	.014	1.035	.017	.017	1.017	.012	.013	.059	-.001		.012	-.006	.106	.039	1.112	.119**	.043	1.126
Gender female	-.122	.190	.885	.056	.228	1.058	-.036	.184	-.012	.079		.158	.027	-.419	.575	.658	-.307	.660	.735
Educational level																			
Low	Ref			Ref			Ref			Ref				Ref			Ref		
Medium	.254	.241	1.289	.282	.290	1.325	-.379	.232	-.104	-.411		.198	-.113*	.377	.666	1.458	.415	.753	1.514
High	.305	.236	1.356	.100	.276	1.105	.026	.240	.008	-.218		.206	-.064	.086	.629	1.090	-.060	.748	.942
Job Status retired (vs. employed)	-.155	.280	.857	.207	.349	1.230	.108	.236	.029	.470		.204	.128*	.699	.825	2.011	.744	.978	2.104
Marital status																			
Married	Ref			Ref			Ref			Ref				Ref			Ref		
Single	-.058	.451	.944	.090	.551	1.094	-.046	.414	-.012	.029		.352	.007	.241	1.323	1.273	.464	1.453	1.590
Widowed	.819	.474	2.268	.884	.593	2.420	.008	.440	.002	.199		.374	.050	1.178	1.225	3.249	1.298	1.356	3.662
Insurance (statutory vs. private)	-.562	.224	.570	-.381	.258	.683	-.133	.206	-.042	-.053		.177	-.017	.250	.623	1.284	.275	.709	1.316
Town size (small vs. large)	.343	.189	1.409	.546*	.226	1.727	.028	.183	.010	.248		.157	.085	.032	.539	1.033	-.439	.599	.645
Living with partner (vs. living alone)	-.480	.438	.619	-.611	.544	.543	-.351	.400	-.113	-.369		.339	-.119	-.514	1.159	.598	-.928	1.230	.395
Having children (vs. not having children)	.304	.267	1.355	.337	.327	1.401	-.024	.255	-.006	.039		.218	.009	1.629	.807	5.097	1.771*	.885	5.875
Physical health	-.239	.115	.787	-.267	.137	.766	.004	.112	.002	.036		.096	.021	-.112	.345	.894	.070	.400	1.072
No chronic disease (vs. chronically ill)	.445	.195	1.560	.502*	.232	1.653	.016	.187	.006	-.026		.160	-.009	.625	.557	1.868	.708	.634	2.031
No recommendation to vaccinate (vs. recommendation)	.710	.223	2.034	.749**	.270	2.115	.475	.192	.152	.363		.164	.116	3.017	.548	20.420	2.766***	.601	15.897
Doctor visit																			
< every 2–3 months	Ref			Ref			Ref			Ref				Ref			Ref		
Every 2–3 months	.578	.207	1.783	.497*	.246	1.644	.067	.200	.022	.038		.171	.013	.690	.583	1.994	.837	.643	2.310
> 3 months	.565	.290	1.759	.328	.340	1.388	.270	.300	.060	.286		.256	.064	.856	.838	2.355	.744	.949	2.104
Duration to reach doctor																			
< 5 min	Ref			Ref			Ref			Ref				Ref			Ref		
6–10 min	-.277	.227	.758	.003	.271	1.003	-.074	.214	-.025	.166		.187	.057	-.545	.654	.580	-.298	.744	.742
> 10 min	-.101	.230	.904	.207	.278	1.230	-.035	.220	-.012	.031		.190	.010	-.047	.664	.954	-.017	.753	.983
Coll. Responsibility				-.002	.112	.998				.087		.068	.066				.393	.316	1.481
Confidence				.723***	.081	2.061				.336		.050	.367***				.266	.251	1.305
Constraints				-.450*	.180	.638				-.098		.101	-.049				-.370	.273	.691
Complacency				-.292**	.086	.747				-.409		.073	-.299***				-.433**	.170	.649
Calculation				-.334***	.069	.716				-.114		.052	-.114*				.013	.208	1.013
Observations	600						298							144					
Cox & Snell *R*^2^ Nagelkerkes *R*^2^	.144 .192			.362 .482			.059			.336				.400 .536			.461 .617		
Model test	$\chi 2$ (18) = 93.355***			$\chi 2$ (23) = 269.398***			*F*(18, 279) =.974, n.s			*F*(23, 274) = 6.018***				$\chi 2$ (18) = 73.657***			$\chi 2$ (23) = 89.028***		

Stepwise regressions. * p <.05, ** *p* <.01, *** *p* <.001. The 5 C psychological antecedents complacency and collective responsibility were recoded. Higher values for complacency indicate that participants are more complacent and feel less at risk to influenza. Higher values for collective responsibility indicate higher prosocial motivation to get vaccinated

Results showed that higher confidence was associated with a greater likelihood of being vaccinated against influenza. Higher complacency, constraints and calculation were associated with being less likely vaccinated against influenza. Results from linear regressions predicting the intention to get vaccinated revealed that higher confidence was associated with an increase, whereas higher complacency and calculation with a decrease in influenza vaccination intention. For pneumococcal vaccination the pattern was quite different: only complacency was significantly associated with being vaccinated against pneumococci, indicating that being more complacent was related to lower probability of being vaccinated.

From the sociodemographic factors, larger town size and being chronically ill were related to more influenza vaccination behavior. From the other factors that have shown to be related to vaccination behavior, more doctor visits and having received an influenza vaccine recommendation from the doctor were associated with a greater probability of being vaccinated against influenza. Being employed was associated with higher influenza vaccine intention whereas having a medium educational level lowered the intention. Increasing age, having children and receiving a recommendation for pneumococcal vaccination was associated with an increase in pneumococcal vaccination behavior.

##### Subnational sample

**Fig. 4:**
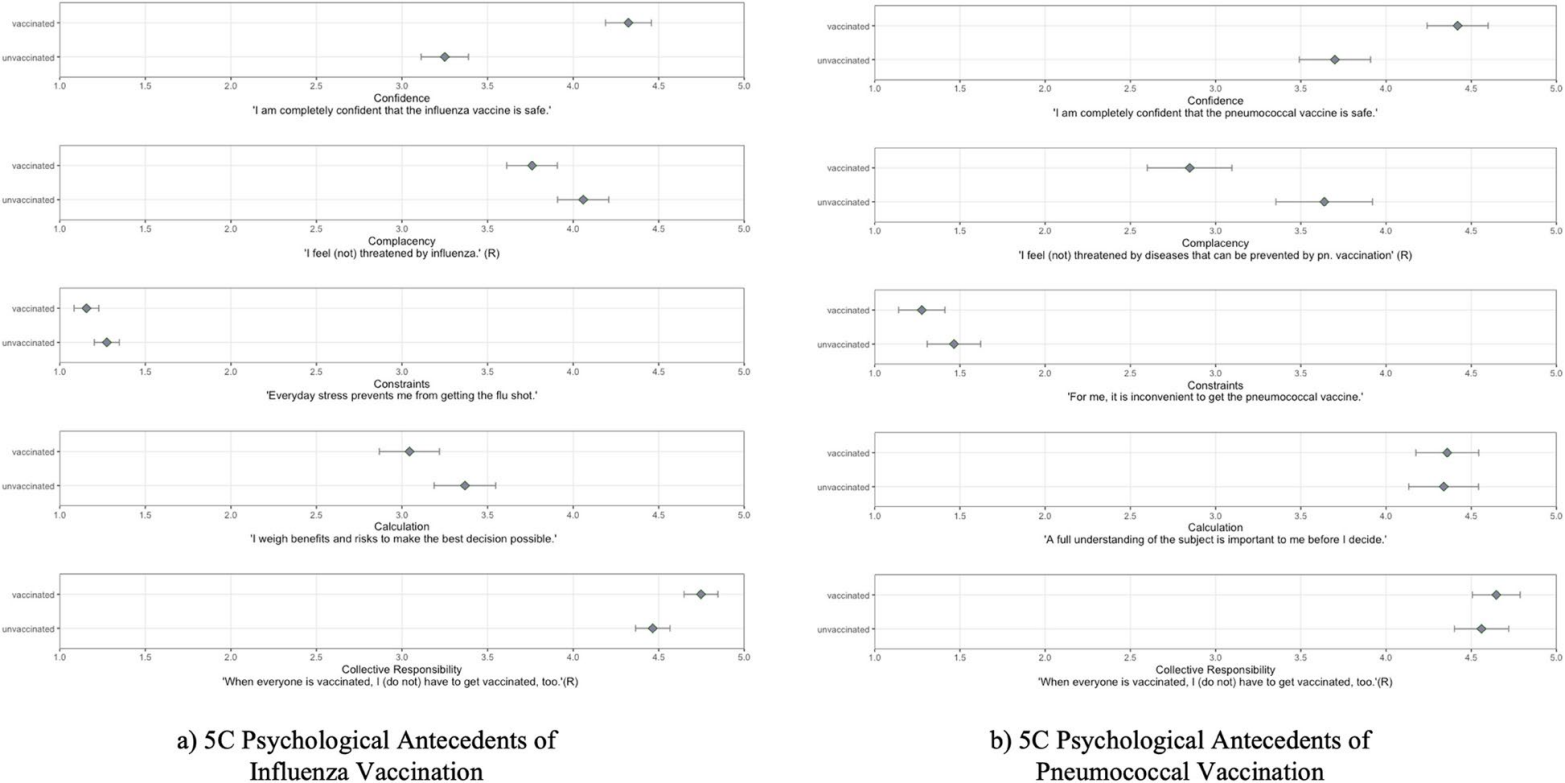
5 C antecedents of vaccination in the subnational sample. *Note.* The Figure shows estimated mean differences and 95% CIs for the 5 C psychological antecedents of vaccination between unvaccinated vs. vaccinated participants. **a** Influenza vaccination. Participants vaccinated against influenza show higher confidence, engage less in the calculation of risks and benefits of influenza vaccination and show higher collective responsibility compared to unvaccinated participants. **b** Pneumococcal vaccination. Participants vaccinated against pneumococci show higher confidence and are less complacent compared to unvaccinated participants.. Results are obtained from the analysis of regression in Table 5

The results are presented in Table 5 and Fig. 4. Including the 5 C psychological antecedents of vaccination again accounted for an additional amount of variance in influenza ($\Delta$ *R*^2^ = 0.172) and pneumococcal ($\Delta$ *R*^2^ = 0.124) vaccination behavior beyond sociodemographic and other factors surrounding vaccination.

**Table 5:** Results of regression analyses for the determinants of vaccination behavior and intention in the subnational sample

	**Influenza Vaccination Behavior**						**Influenza Vaccination Intention**						**Pneumococcal Vaccination Behavior**					
	**Step 1**			**Step 2**			**Step 1**			**Step 2**			**Step 1**			**Step 2**		
**Determinants**	**B**	**SE**	**OR**	**B**	**SE**	**OR**	**B**	**SE**	${\varvec{\upbeta}}$	**B**	**SE**	${\varvec{\upbeta}}$	**B**	**SE**	**OR**	**B**	**SE**	**OR**
(Constant)	− 3.833	1.572	.022	− 6.360	1.854	.002	2.840	1.764		1.092	1.663		− 8.965	3.256	.000	− 9.101	3.783	.000
Age	.055***	.014	1.057	.050**	.015	1.051	.010	.016	.041	.014	.015	.057	.044	.029	1.045	.027	.032	1.028
Gender female	-.327	.196	.721	-.170	.213	.843	-.377	.237	-.098	-.245	.215	-.064	-.133	.404	.875	.085	.449	1.089
Educational level																		
Low	Ref			Ref			Ref			Ref			Ref			Ref		
Medium	-.028	.245	.972	-.043	.265	.958	-.559*	.281	-.127	-.476	.253	-.108	.180	.470	1.197	-.035	.533	.965
High	.287	.213	1.333	.333	.233	1.396	-.490	.261	-.124	-.458	.236	-.115	-.307	.428	.736	-.099	.473	.906
Job Status retired (vs. employed)	-.341	.282	.711	-.364	.308	.695	− 1.068	.290	-.232	-.949	.266	-.206***	-.042	.571	.959	-.547	.624	.579
Marital status																		
Married	Ref			Ref			Ref			Ref			Ref			Ref		
Single	-.359	.462	.698	-.187	.513	.829	-.592	.489	-.114	-.372	.443	-.071	− 1.094	1.011	.335	− 1.309	1.028	.270
Widowed	-.151	.466	.859	.079	.517	1.082	-.858	.488	-.202	-.694	.447	-.163	-.717	1.014	.488	− 1.247	1.038	.287
Insurance (statutory vs. private)	-.315	.398	.730	-.356	.438	.700	-.103	.426	-.015	-.044	.385	-.006	-.029	.825	.971	-.206	.918	.814
Town size (small vs. large)	.355	.186	1.427	.406*	.204	1.501	.237	.213	.066	.241	.192	.068	1.033	.399	2.811	1.247**	.453	3.480
Living with partner (vs. living alone)	-.176	.456	.839	-.643	.507	.526	.464	.464	.122	.095	.423	.025	.988	.968	2.685	1.435	.966	4.199
Having children (vs. not having children)	-.473	.372	.623	-.241	.405	.786	-.172	.387	-.027	.013	.350	.002	.933	1.115	2.543	.931	1.174	2.538
Physical health	.011	.115	1.011	-.044	.125	.957	-.139	.137	-.069	-.161	.124	-.080	-.074	.236	.928	-.189	.277	.828
No chronic disease (vs. chronically ill)	.059	.203	1.061	.088	.223	1.092	.133	.239	.037	.121	.217	.034	-.739	.415	.478	-.954*	.467	.385
No recommendation to vaccinate (vs. recommendation)	.385	.285	1.470	.396	.314	1.496	.422	.303	.082	.477	.273	.093	2.522	.385	12.452	2.604***	.429	13.518
Doctor visit																		
< every 2–3 months	Ref			Ref			Ref			Ref			Ref			Ref		
Every 2–3 months	.746**	.223	2.109	.651**	.246	1.917	.513*	.250	.144	.272	.226	.076	.435	.441	1.544	.404	.490	1.498
> 3 months	.841**	.274	2.318	.623*	.302	1.865	.406	.317	.091	.192	.286	.043	.259	.544	1.295	.018	.614	1.018
Duration to reach doctor																		
< 5 min	Ref			Ref			Ref			Ref			Ref			Ref		
6–10 min	.221	.234	1.248	.171	.258	1.186	.037	.276	.010	.045	.251	.012	.119	.474	1.127	-.133	.541	.875
> 10 min	-.263	.240	.768	-.283	.264	.753	.071	.273	.020	.061	.248	.017	-.330	.476	.719	-.481	.564	.618
Coll. Responsibility				.348**	.113	1.416				.191	.087	.120*				.274	.248	1.316
Confidence				.607***	.083	1.835				.420	.062	.360***				.671***	.199	1.957
Constraints				-.163	.156	.849				-.042	.132	-.017				-.283	.255	.753
Complacency				-.064	.070	.938				-.224	.074	-.171***				-.342*	.134	.710
Calculation				-.119*	.059	.888				.018	.058	.017				-.208	.206	.812
Observations	590						281						218					
Cox & Snell *R*^2^ Nagelkerkes *R*^2^	.115 .153			.244 .325			.153			.178			.313 .421			.406 .545		
Model test	$\chi 2$(18) = 71.794***			$\chi 2$(23) = 164.779***			*F*(18, 262) = 2.630***			*F*(18, 257) = 5.520***			$\chi 2$(18) = 81.833***			$\chi 2$(23) = 113.445***		

Stepwise regressions. * p <.05, ** *p* <.01, *** *p* <.001. The 5 C psychological antecedents complacency and collective responsibility were recoded. Higher values for complacency indicate that participants are more complacent and feel less at risk to influenza. Higher values for collective responsibility indicate higher prosocial motivation to get vaccinated

In the subnational sample, higher confidence was related to an increase and calculation to a decrease in influenza vaccination behavior. Moreover, collective responsibility was related to increased vaccination behavior. Higher collective responsibility and higher confidence were related to increased influenza vaccination intention whereas higher complacency lowered the vaccination intention. Higher confidence was associated with increased pneumococcal vaccination behavior, higher complacency with decreased behavior.

Increasing age, town size and doctor visits were related to increased influenza vaccination behavior, being employed to decreased influenza vaccination intention. Larger town size and receiving a recommendation for pneumococcal vaccination was associated with increased pneumococcal vaccination behavior whereas being chronically ill with decreased behavior.

## Discussion

The results reported in this article show unique patterns in the psychological antecedents for two different samples and two vaccinations: Regarding influenza vaccination, confidence and calculation were important predictors for behavior in a nationally representative and a subnational sample, complacency and constraints were also important in the national sample, and collective responsibility was relevant in the subnational sample. Regarding pneumococcal vaccination, complacency explained behavior in both samples; in addition, confidence also played a role in the subnational sample. The results show that the amount of explained variance in vaccination behavior nearly doubles when psychological determinants of vaccine hesitancy are taken into account.

In this study, we applied the 5 C psychological antecedents of vaccination to examine behavior alongside sociodemographic and system-related factors (e.g., doctor recommendations, visit frequency). Existing frameworks explaining vaccination behavior often combine perceived or physical barriers with psychological determinants.

For example, the COM-B model suggests that vaccination behavior is influenced by Capability, Opportunity, and Motivation [34]. Capability encompasses psychological (e.g., knowledge) and physical (e.g., access) factors, Opportunity refers to external factors like vaccine accessibility and social norms, and Motivation involves both deliberate decision-making and automatic influences such as emotions and habits. For example, a study using the COM-B model among older U.S. adults found that 91.3% were willing to vaccinate, with confidence as a key predictor [35]. Many hesitant individuals relied on their healthcare providers for guidance, reinforcing the idea that both psychological beliefs and external influences—such as provider recommendations and public messaging—play a crucial role in vaccine decision-making.

The BeSD (Behavioral and Social Drivers) framework posits that vaccination behavior is influenced by modifiable beliefs and experiences across four key areas: attitudes toward vaccines, social influences, motivation or hesitancy, and practical access issues [36]. For instance, a study applying the BeSD framework in Italy on influenza vaccination found that, despite free vaccines for high-risk groups, one-third of eligible individuals remained unvaccinated, particularly among seniors and professionals such as teachers and healthcare workers [37]. Barriers included unawareness of being in a target group, concerns about vaccine safety, lower education levels, rural residency, and influence from vaccine-hesitant peers. These findings suggest that raising awareness and addressing misinformation could significantly improve vaccine uptake, particularly among hesitant groups.

The Health Belief Model (HBM) suggests that vaccination decisions are influenced by perceived severity and susceptibility to a disease, as well as the benefits and barriers of vaccination [38, 39]. It also considers cues to action (e.g., reminders or recommendations) and self-efficacy (confidence in one's ability to get vaccinated). Studies applying HBM find that perceived barriers, such as concerns about vaccine safety, are major predictors of hesitancy, while perceived benefits, susceptibility, and cues to action are associated with higher willingness to vaccinate. A systematic review of 16 studies (30,242 participants) found that 33.2% of people were hesitant about COVID- 19 vaccines, with gender, education, income, and prior flu vaccination influencing hesitancy [40]. Similarly, a study in China involving 1,212 elderly participants showed that awareness of vaccine effectiveness and cues to action significantly increased vaccination intention, with health beliefs acting as mediators [41]. In Egypt, only 46.9% of older adults were willing to vaccinate, but those with higher perceived severity, vaccine benefits, and action cues were more likely to accept the vaccine [42]. These findings emphasize the importance of addressing concerns, enhancing awareness, and utilizing social and structural cues to improve vaccine uptake.

Across all models (COM-B, BeSD, and HBM), psychological beliefs about vaccines (such as perceived safety and effectiveness) play a central role in vaccination decisions. Studies consistently show that individuals with higher confidence in vaccine safety and benefits are more likely to get vaccinated, whereas concerns about safety and misinformation drive hesitancy. Additionally, external factors, such as social norms, provider recommendations, and public messaging, influence vaccination behavior in all frameworks. Each model places different emphasis on specific drivers of vaccine uptake. When combining all these findings, a comprehensive view emerges: vaccine uptake is influenced by a mix of individual beliefs, social pressures, and structural barriers. While psychological attitudes (such as trust in vaccine safety) are critical, opportunity and external influences (such as healthcare provider recommendations, social norms, and ease of access) also shape behavior significantly. Addressing hesitancy requires a multi-faceted approach—correcting misinformation, increasing awareness, ensuring access, and leveraging trusted sources like healthcare providers to encourage vaccination among hesitant groups.”

There is a similar pattern for the 5 C on vaccination intentions compared to behavior, however, constraints was not a significant predictor for vaccination intentions in the national sample and complacency significantly predicted intentions in the subnational sample (whereas calculation did not). We can only speculate whether this gap in intention vs. behavior for constraints could actually indicate a particular need for intervention: because it does not predict intentions, it is not anticipated despite its importance for later behavior. More research is needed to test whether targeting vaccination information to the needs at specific stages of individuals’ decision making process can explain differences in behaviors vs. intentions.

The results reveal a pattern of meaningful factors influencing vaccination that can be used to prioritize decision making for intervention designers. For example, the results show that the recommendation from a doctor to get vaccinated influences the behavior in both samples; however the predictive power is even stronger for pneumococcal (*B* = 2.766, *OR* = 15.897, *p* < 0.001) compared to influenza vaccination (*B* = 0.749, *OR* = 2.115, *p* < 0.01). At the same time, the results showed that awareness of the pneumococcal vaccination is still low and only 24.3% of participants received a recommendation to vaccinate against pneumococci compared to 74.3% for influenza (however, percentages are higher in the subnational sample). These findings are in line with another study on pneumococcal vaccination in Germany [24] and indicate that older adults’ awareness of pneumococcal vaccination – and possibly behavior – can be effectively increased by a doctors’ recommendation. Interventions should therefore not only target patients but doctors, too.

Higher age increased influenza vaccination behavior in the subnational sample and pneumococcal vaccination behavior in the national sample, which suggests that the older the participants are, the better vaccinated they are. An explanation could be that it might take a while for individuals to process and understand and actually hear about why these vaccinations are important to prevent infectious diseases. Moreover, illness becomes more prevalent with increasing age which in turn could increase the perceived vulnerability to infections. Routine checkups should include a vaccination status screening and vaccination recommendations as soon as individuals enter this age group to inform them as early as possible.

As a practical implication of this study, the data can be used to design interventions to change vaccination behavior. For example, the results of the subnational survey have already been used in a prospective intervention called Vaccination60 + which aimed at increasing influenza and pneumococcal vaccination behavior in older adults in the region of Thuringia in Germany between 2016–2019. Calculation as a determinant of vaccination behavior lead to the choice of an educational campaign design to support the decision making process. Confidence, collective responsibility and complacency were identified to design the content of the information campaign. Findings regarding influenza knowledge informed the decision to implement the *debunking* approach which is defined as “presenting a corrective message that establishes that the prior message was misinformation” [43]. It was used to counter the identified misconceptions relevant to vaccination behavior.

The findings presented here have some limitations. For instance, we only assessed the 5 C antecedents of pneumococcal vaccination in participants who heard about pneumococcal disease before, this filter strategy reduced our sample in the respective analyses. However, the findings are in line with other work in this field and together they indicate that more behavioral interventions are needed to increase pneumococcal vaccination.

We assessed the data cross-sectionally which does not allow to draw a causal conclusion. To ensure robust evidence for behavioral differences and their association with psychological determinants, the surveys were powered for medium effect sizes, and the sample was quota distributed to be representative of the German population. By integrating our findings into the international literature, conducting missing data analyses and imputations, and utilizing a low-barrier telephone survey method, we are confident in assessing our data as robust.

## Conclusions

In sum, sociodemographic factors alone cannot explain vaccination behavior well: even if the same factors surrounding vaccination were relevant (recommendation, age) for influenza and pneumococcal vaccination, the psychological profiles for vaccination were different for the two different vaccinations. This suggests that interventions to increase vaccine uptake need to take different aspects into account – depending on the vaccine. The data presented in this article allows the following recommendations for future interventions: (i) target doctors in interventions for pneumococcal vaccination, support them on how they can inform patients and increase awareness about pneumococcal vaccination; (ii) target misperceptions about influenza vaccination with evidence-based communication approaches, e.g. debunking; (iii) assess psychological profiles before the development of an intervention because this will help to focus on the relevant factors; (iv) and target older adults as soon as they enter the age group for vaccination.

The need for more data on pneumococcal vaccination intention and behavior remains critical to address existing gaps in the literature. As Nasreen et al. (2022) emphasize in their scoping review, further research in this area is still essential to better understand the factors that influence vaccination decisions and to develop more effective interventions [44].

Since the COVID- 19 pandemic has increased the awareness for vaccination as prevention, but may have raised may questions and some doubts in the public, doctors could take advantage of this moment to inform patients about all the options available to fight life-threatening infectious diseases.

## Supplementary Information

Supplementary Material 1.

## Data Availability

The dataset supporting the conclusions is available in the Open Science Framework repository (private, view-only link for review): https://osf.io/8my5k/.
