# Supplementary material for: Influenza and pneumococcal vaccine hesitancy in the elderly population: results from two representative surveys in Germany
Source: BMC Public Health. 2025 May 6;25:1672. doi: 10.1186/s12889-025-22441-9 (PMC12054266; doi:10.1186/s12889-025-22441-9)
Supplement: Supplementary file 1 — Supplementary Material 1. [file 12889_2025_22441_MOESM1_ESM.docx]

## **Supplement Part I - Missing Data Analysis**

First, we can assess whether individuals included in the analysis differ significantly from those not included, based on any of the analysis variables.

1. Data Set for **Flu Uptake Subnational**
   Comparing n = 590 participants in the analysis with n = 110 people missing in the analysis. The results show that there are no significant differences between the drop-outs and the included participants. Due to the high number of tests, a Bonferroni correction was also made for the alpha value. Here, too, there are no significant differences between included participants and drop-outs on the categorical variables.

### Table S1 Chi-Squared Results

| Variable | *p* | *p* adjusted |
| --- | --- | --- |
| Gender (male vs. female) | 0.4184 | 1 |
| Education = medium (vs. low) | 0.8188 | 1 |
| Education = high (vs. low) | 0.2168 | 1 |
| Jobstatus = working (vs. retired) | 0.6005 | 1 |
| Marital status = single (vs. married) | 0.1012 | 1 |
| Marital status = widowed (vs. married) | 0.3953 | 1 |
| Insurance = statutory (vs. private) | 0.9620 | 1 |
| Town size (small vs. large) | 0.7672 | 1 |
| Living with partner (vs. living alone) | 0.3978 | 1 |
| Having children (vs. not having children) | 0.1342 | 1 |
| No Chronic Diseases (vs. yes) | 0.4331 | 1 |
| No recommendation to vaccinate (vs. rec by GP) | 0.4446 | 1 |
| Doc. visit = Every 2-3 months (vs. <2-3 months) | 1.0000 | 1 |
| Doc. visit = > 3 months (vs. <2-3 months) | 0.7877 | 1 |
| Duration to reach doc = 6-10 min (vs. <5 min) | 0.9115 | 1 |
| Duration to reach doc = >10 min (vs. <5 min) | 0.0779 | 1 |

*Note*. Adjusted p-Values using Bonferroni Correction for this comparison set.

1. Data Set for **Flu Intention Subnational**Comparing n = 281 participants in the analysis with n = 419 people missing in the analysis. The results show that there are no significant differences between the drop-outs and the included participants, except for highest education, jobstatus and frequency of doctoral visits. Due to the high number of tests, a Bonferroni correction was also made for the alpha value. Here, there are no significant differences between included participants and drop-outs on the categorical variables.

### Table S2 Chi-Squared Results

| Variable | *p* | *p* adjusted |
| --- | --- | --- |
| Gender (male vs. female) | 0.0099 | 0.1790 |
| Education = medium (vs. low) | 0.3445 | 1.0000 |
| Education = high (vs. low) | **0.0114** | 0.2049 |
| Jobstatus = working (vs. retired) | **0.0031** | 0.0551 |
| Marital status = single (vs. married) | 0.4043 | 1.0000 |
| Marital status = widowed (vs. married) | 0.2774 | 1.0000 |
| Insurance = statutory (vs. private) | 0.2970 | 1.0000 |
| Town size (small vs. large) | 0.1157 | 1.0000 |
| Living with partner (vs. living alone) | 0.9753 | 1.0000 |
| Having children (vs. not having children) | 0.6712 | 1.0000 |
| No Chronic Diseases (vs. yes) | 0.2465 | 1.0000 |
| No recommendation to vaccinate (vs. rec by GP) | 0.3086 | 1.0000 |
| Doc. visit = Every 2-3 months (vs. <2-3 months) | **0.0163** | 0.2926 |
| Doc. visit = > 3 months (vs. <2-3 months) | **0.0359** | 0.6470 |
| Duration to reach doc = 6-10 min (vs. <5 min) | 0.1037 | 1.0000 |
| Duration to reach doc = >10 min (vs. <5 min) | 0.1849 | 1.0000 |

*Note*. Adjusted p-Values using Bonferroni Correction for this comparison set.

1. Data Set for **Flu Uptake National**
   Comparing n = 600 participants in the analysis with n = 101 people missing in the analysis. The results show that there are no significant differences between the drop-outs and the included participants, except the recommendation of vaccination by a GP. Due to the high number of tests, a Bonferroni correction was also made for the alpha value. Here, there are no significant differences between included participants and drop-outs on the categorical variables.

### Table S3 Chi-Squared Results

| Variable | *p* | *p* adjusted |
| --- | --- | --- |
| Gender (male vs. female) | 0.4736 | 1.0000 |
| Education = medium (vs. low) | 0.3383 | 1.0000 |
| Education = high (vs. low) | 0.8338 | 1.0000 |
| Jobstatus = working (vs. retired) | 0.9419 | 1.0000 |
| Marital status = single (vs. married) | 0.3582 | 1.0000 |
| Marital status = widowed (vs. married) | 0.4906 | 1.0000 |
| Insurance = statutory (vs. private) | 0.3023 | 1.0000 |
| Town size (small vs. large) | 0.5740 | 1.0000 |
| Living with partner (vs. living alone) | 0.6738 | 1.0000 |
| Having children (vs. not having children) | 1.0000 | 1.0000 |
| No Chronic Diseases (vs. yes) | 0.1110 | 1.0000 |
| No recommendation to vaccinate (vs. rec by GP) | **0.0391** | 0.7429 |
| Doc. visit = Every 2-3 months (vs. <2-3 months) | 0.0574 | 1.0000 |
| Doc. visit = > 3 months (vs. <2-3 months) | 0.5634 | 1.0000 |
| Duration to reach doc = 6-10 min (vs. <5 min) | 0.1637 | 1.0000 |
| Duration to reach doc = >10 min (vs. <5 min) | 0.2790 | 1.0000 |
| Fed. State | 0.0874 | 1.0000 |

*Note*. Adjusted p-Values using Bonferroni Correction for this comparison set.

1. Data Set for **Flu Intention National**Comparing n = 298 participants in the analysis with n = 403 people missing in the analysis. The results show that there are few significant differences between the drop-outs and the included participants. Therefore, Table S4a shows the distributions per variable. Due to the high number of tests, a Bonferroni correction was also made for the alpha values. Here, the remaining significant differences show less chronic diseases, less recommendations for vaccines from the GP, and higher frequency of doctoral visits (rather every 2-3months than less often).

### Table S4 Chi-Squared Results

| Variable | *p* | *p* adjusted |
| --- | --- | --- |
| Gender (male vs. female) | 0.5718 | 1.0000 |
| Education = medium (vs. low) | 0.3452 | 1.0000 |
| Education = high (vs. low) | 0.6994 | 1.0000 |
| Jobstatus = working (vs. retired) | **0.0033** | 0.0585 |
| Marital status = single (vs. married) | 0.1536 | 1.0000 |
| Marital status = widowed (vs. married) | **0.0045** | 0.0809 |
| Insurance = statutory (vs. private) | 0.0594 | 1.0000 |
| Town size (small vs. large) | 0.0537 | 0.9669 |
| Living with partner (vs. living alone) | 0.3544 | 1.0000 |
| Having children (vs. not having children) | 0.6182 | 1.0000 |
| No Chronic Diseases (vs. yes) | **0.0003** | **0.0056** |
| No recommendation to vaccinate (vs. rec by GP) | **0.0014** | **0.0252** |
| Doc. visit = Every 2-3 months (vs. <2-3 months) | **0.0019** | **0.0340** |
| Doc. visit = > 3 months (vs. <2-3 months) | **0.0276** | 0.4962 |
| Duration to reach doc = 6-10 min (vs. <5 min) | 0.6526 | 1.0000 |
| Duration to reach doc = >10 min (vs. <5 min) | 0.6710 | 1.0000 |
| Fed. State | **0.0001** | **0.0017** |

*Note*. Adjusted p-Values using Bonferroni Correction for this comparison set.

### Table S4a – Distributional differences in the included vs. drop-out samples

| **Variable** | **Category** | **drop-out** | **included** |
| --- | --- | --- | --- |
| Gender | male | 187 (46.4%) | 131 (44.0%) |
|  | female | 216 (53.6%) | 167 (56.0%) |
| Age Group | 60-69 | 136 (33.7%) | 154 (51.7%) |
|  | 70-79 | 171 (42.4%) | 107 (35.9%) |
|  | 80-89 | 82 (20.3%) | 35 (11.7%) |
|  | 90-99 | 14 (3.5%) | 2 (0.7%) |
|  | 100+ | 0 (0.0%) | 0 (0.0%) |
| Education = medium (vs. low) | 0 | 299 (74.2%) | 240 (80.5%) |
|  | 1 | 88 (21.8%) | 58 (19.5%) |
|  | NA | 16 (4.0%) | 0 (0.0%) |
| Education = high (vs. low) | 0 | 290 (72.0%) | 228 (76.5%) |
|  | 1 | 97 (24.1%) | 70 (23.5%) |
|  | NA | 16 (4.0%) | 0 (0.0%) |
| Jobstatus = working (vs. retired) | 0 | 360 (89.3%) | 242 (81.2%) |
|  | 1 | 43 (10.7%) | 56 (18.8%) |
| Marital status = single (vs. married) | 0 | 348 (86.4%) | 249 (83.6%) |
|  | 1 | 49 (12.2%) | 49 (16.4%) |
|  | NA | 6 (1.5%) | 0 (0.0%) |
| Marital status = widowed | 0 | 298 (73.9%) | 251 (84.2%) |
|  | 1 | 99 (24.6%) | 47 (15.8%) |
|  | NA | 6 (1.5%) | 0 (0.0%) |
| Insurance = statutory | 0 | 308 (76.4%) | 211 (70.8%) |
|  | 1 | 90 (22.3%) | 87 (29.2%) |
|  | NA | 5 (1.2%) | 0 (0.0%) |
| Town size = small | 0 | 143 (35.5%) | 128 (43.0%) |
|  | 1 | 260 (64.5%) | 170 (57.0%) |
| Living with partner | 0 | 258 (64.0%) | 205 (68.8%) |
|  | 1 | 138 (34.2%) | 93 (31.2%) |
|  | NA | 7 (1.7%) | 0 (0.0%) |
| Having children | 0 | 337 (83.6%) | 256 (85.9%) |
|  | 1 | 63 (15.6%) | 42 (14.1%) |
|  | NA | 3 (0.7%) | 0 (0.0%) |
| Chronic Diseases | 0 | 163 (40.4%) | 163 (54.7%) |
|  | 1 | 238 (59.1%) | 135 (45.3%) |
|  | NA | 2 (0.5%) | 0 (0.0%) |
| Received Recommendation Flu | 0 | 77 (19.1%) | 91 (30.5%) |
|  | 1 | 314 (77.9%) | 207 (69.5%) |
|  | NA | 12 (3.0%) | 0 (0.0%) |
| Doc. visit = Every 2-3 months (vs. <2-3 months) | 0 | 214 (53.1%) | 194 (65.1%) |
|  | 1 | 189 (46.9%) | 104 (34.9%) |
| Doc. visit = > 3 months (vs. <2-3 months) | 0 | 330 (81.9%) | 263 (88.3%) |
|  | 1 | 73 (18.1%) | 35 (11.7%) |
| Duration to reach doc = 6-10 min (vs. <5 min) | 0 | 250 (62.0%) | 179 (60.1%) |
|  | 1 | 153 (38.0%) | 119 (39.9%) |
| Duration to reach doc = > 10 min (vs. <5 min) | 0 | 259 (64.3%) | 197 (66.1%) |
|  | 1 | 144 (35.7%) | 101 (33.9%) |
| Federal State | SH | 9 (2.2%) | 14 (4.7%) |
|  | Hamburg | 16 (4.0%) | 9 (3.0%) |
|  | NDS | 45 (11.2%) | 36 (12.1%) |
|  | HB | 9 (2.2%) | 1 (0.3%) |
|  | NRW | 64 (15.9%) | 55 (18.5%) |
|  | Hessen | 22 (5.5%) | 25 (8.4%) |
|  | RLP | 19 (4.7%) | 15 (5.0%) |
|  | BW | 51 (12.7%) | 31 (10.4%) |
|  | Bayern | 32 (7.9%) | 52 (17.4%) |
|  | Saarland | 6 (1.5%) | 8 (2.7%) |
|  | Berlin | 27 (6.7%) | 16 (5.4%) |
|  | BB | 18 (4.5%) | 4 (1.3%) |
|  | MVP | 18 (4.5%) | 5 (1.7%) |
|  | Sachsen | 33 (8.2%) | 17 (5.7%) |
|  | S-Anhalt | 17 (4.2%) | 4 (1.3%) |
|  | Thüringen | 17 (4.2%) | 6 (2.0%) |
|  | Total | 403 (100.0%) | 298 (100.0%) |

Interpretation: The participants who have already been vaccinated during the time of the survey could not be considered for their prospective vaccine intention. As those who have already been vaccinated were those highly affected by chronical conditions and under close monitoring by their GPs, those differences emerge in the drop-out analysis as well.

1. Data Set for **Pneu Uptake Subnational**
   Comparing n = 218 participants in the analysis with n = 482 people missing in the analysis.

### Table S5 Chi-Squared Results

| Variable | *p* | *p* adjusted |
| --- | --- | --- |
| Gender (male vs. female) | **0.0368** | 0.6987 |
| Education = medium (vs. low) | 0.0529 | 1.0000 |
| Education = high (vs. low) | **0.0307** | 0.5840 |
| Jobstatus = working (vs. retired) | 1.0000 | 1.0000 |
| Marital status = single (vs. married) | 0.1191 | 1.0000 |
| Marital status = widowed (vs. married) | 1.0000 | 1.0000 |
| Insurance = statutory (vs. private) | 0.8656 | 1.0000 |
| Town size (small vs. large) | 0.3167 | 1.0000 |
| Living with partner (vs. living alone) | 0.9259 | 1.0000 |
| Having children (vs. not having children) | 0.0699 | 1.0000 |
| No Chronic Diseases (vs. yes) | 0.1816 | 1.0000 |
| No recommendation to vaccinate (vs. rec by GP) | **0.0000** | **0.0000** |
| Doc. visit = Every 2-3 months (vs. <2-3 months) | 0.8027 | 1.0000 |
| Doc. visit = > 3 months (vs. <2-3 months) | 0.6022 | 1.0000 |
| Duration to reach doc = 6-10 min (vs. <5 min) | 1.0000 | 1.0000 |
| Duration to reach doc = >10 min (vs. <5 min) | 0.9253 | 1.0000 |

*Note*. Adjusted p-Values using Bonferroni Correction for this comparison set.

### Table S5a - Distributional differences in the included vs. drop-out samples

| Variable |  | drop-out | included |
| --- | --- | --- | --- |
| Gender | male | 187 (38.8%) | 66 (30.3%) |
|  | female | 295 (61.2%) | 152 (69.7%) |
|  | Total | 482 (100.0%) | 218 (100.0%) |
| Age Group | 60-69 | 201 (41.7%) | 91 (41.7%) |
|  | 70-79 | 176 (36.5%) | 88 (40.4%) |
|  | 80-89 | 98 (20.3%) | 38 (17.4%) |
|  | 90-99 | 7 (1.5%) | 1 (0.5%) |
|  | 100+ | 0 (0.0%) | 0 (0.0%) |
|  | Total | 482 (100.0%) | 218 (100.0%) |
| Education = medium (vs. low) | 0 | 388 (80.5%) | 169 (77.5%) |
|  | 1 | 74 (15.4%) | 49 (22.5%) |
|  | NA | 20 (4.1%) | 0 (0.0%) |
|  | Total | 482 (100.0%) | 218 (100.0%) |
| Education = high (vs. low) | 0 | 324 (67.2%) | 134 (61.5%) |
|  | 1 | 138 (28.6%) | 84 (38.5%) |
|  | NA | 20 (4.1%) | 0 (0.0%) |
|  | Total | 482 (100.0%) | 218 (100.0%) |
| Jobstatus = working (vs. retired) | 0 | 418 (86.7%) | 190 (87.2%) |
|  | 1 | 63 (13.1%) | 28 (12.8%) |
|  | NA | 1 (0.2%) | 0 (0.0%) |
|  | Total | 482 (100.0%) | 218 (100.0%) |
| Marital status = single (vs. married) | 0 | 409 (84.9%) | 199 (91.3%) |
|  | 1 | 62 (12.9%) | 19 (8.7%) |
|  | NA | 11 (2.3%) | 0 (0.0%) |
|  | Total | 482 (100.0%) | 218 (100.0%) |
| Marital status = widowed | 0 | 356 (73.9%) | 165 (75.7%) |
|  | 1 | 115 (23.9%) | 53 (24.3%) |
|  | NA | 11 (2.3%) | 0 (0.0%) |
|  | Total | 482 (100.0%) | 218 (100.0%) |
| Insurance = statutory | 0 | 447 (92.7%) | 207 (95.0%) |
|  | 1 | 27 (5.6%) | 11 (5.0%) |
|  | NA | 8 (1.7%) | 0 (0.0%) |
|  | Total | 482 (100.0%) | 218 (100.0%) |
| Town size = small | 0 | 191 (39.6%) | 77 (35.3%) |
|  | 1 | 291 (60.4%) | 141 (64.7%) |
|  | Total | 482 (100.0%) | 218 (100.0%) |
| Living with partner | 0 | 323 (67.0%) | 151 (69.3%) |
|  | 1 | 148 (30.7%) | 67 (30.7%) |
|  | NA | 11 (2.3%) | 0 (0.0%) |
|  | Total | 482 (100.0%) | 218 (100.0%) |
| Having children (vs. not  having children) | 0 | 434 (90.0%) | 208 (95.4%) |
|  | 1 | 42 (8.7%) | 10 (4.6%) |
|  | NA | 6 (1.2%) | 0 (0.0%) |
|  | Total | 482 (100.0%) | 218 (100.0%) |
| No Chronic Diseases (vs.  yes) | 0 | 188 (39.0%) | 74 (33.9%) |
|  | 1 | 287 (59.5%) | 144 (66.1%) |
|  | NA | 7 (1.5%) | 0 (0.0%) |
|  | Total | 482 (100.0%) | 218 (100.0%) |
| No recommendation to  vaccinate (vs. Pneomococcal vaccine  recommendation) | 0 | 60 (12.4%) | 22 (10.1%) |
|  | 1 | 415 (86.1%) | 196 (89.9%) |
|  | NA | 7 (1.5%) | 0 (0.0%) |
|  | Total | 482 (100.0%) | 218 (100.0%) |
| Doc. visit = Every 2-3 months (vs. <2-3 months) | 0 | 251 (52.1%) | 112 (51.4%) |
|  | 1 | 225 (46.7%) | 106 (48.6%) |
|  | NA | 6 (1.2%) | 0 (0.0%) |
|  | Total | 482 (100.0%) | 218 (100.0%) |
| Doc. visit = > 3 months (vs. <2-3 months) | 0 | 361 (74.9%) | 170 (78.0%) |
|  | 1 | 115 (23.9%) | 48 (22.0%) |
|  | NA | 6 (1.2%) | 0 (0.0%) |
|  | Total | 482 (100.0%) | 218 (100.0%) |
| Duration to reach doc = 6-10 min (vs. <5 min) | 0 | 275 (57.1%) | 129 (59.2%) |
|  | 1 | 190 (39.4%) | 89 (40.8%) |
|  | NA | 17 (3.5%) | 0 (0.0%) |
|  | Total | 482 (100.0%) | 218 (100.0%) |
| Duration to reach doc = > 10 min (vs. <5 min) | 0 | 287 (59.5%) | 133 (61.0%) |
|  | 1 | 178 (36.9%) | 85 (39.0%) |
|  | NA | 17 (3.5%) | 0 (0.0%) |
|  | Total | 482 (100.0%) | 218 (100.0%) |

1. Data Set for **Pneu Uptake National**
   Comparing n = 144 participants in the analysis with n = 557 people missing in the analysis.

### Table S6 Chi-Squared Results

| Variable | *p* | *p* adjusted |
| --- | --- | --- |
| Gender (male vs. female) | 0.0975 | 1.0000 |
| Education = medium (vs. low) | 0.3371 | 1.0000 |
| Education = high (vs. low) | 0.6012 | 1.0000 |
| Jobstatus = working (vs. retired) | 0.3030 | 1.0000 |
| Marital status = single (vs. married) | 0.4507 | 1.0000 |
| Marital status = widowed (vs. married) | 0.3889 | 1.0000 |
| Insurance = statutory (vs. private) | 0.5025 | 1.0000 |
| Town size (small vs. large) | 0.0784 | 1.0000 |
| Living with partner (vs. living alone) | 0.1399 | 1.0000 |
| Having children (vs. not having children) | 0.9662 | 1.0000 |
| No Chronic Diseases (vs. yes) | 0.9490 | 1.0000 |
| No recommendation to vaccinate (vs. rec by GP) | **0.0000** | **0.0000** |
| Doc. visit = Every 2-3 months (vs. <2-3 months) | 0.3140 | 1.0000 |
| Doc. visit = > 3 months (vs. <2-3 months) | 0.3399 | 1.0000 |
| Duration to reach doc = 6-10 min (vs. <5 min) | 0.2037 | 1.0000 |
| Duration to reach doc = >10 min (vs. <5 min) | 0.8710 | 1.0000 |

*Note*. Adjusted p-Values using Bonferroni Correction for this comparison set.

## **Supplement Part II - Imputation by Means, Imputation by Modus**

To address the issue of missing data, we imputed missing values to ensure the dataset remained suitable for analysis without discarding valuable information. For continuous or interval-scale variables, we imputed missing numerical values by replacing them with the mean of the observed values for that specific variable (imputation by means). For categorical variables, we imputed missing values by replacing them with the most frequently occurring category, or the mode, within that variable (imputation by mode). For binary (dummy) variables, we handled missing values using imputation by mode as well.

Assumption tests covered Cooks’ distances, VIFs and AICs. All assumption test results can be found in the OSF supplement. Table S7 shows maximum values for the assumption tests for the regression analyses.
Table S7 – Assumption Tests

| **Analysis** | **Cooks Distance (max)** | **VIFs (max)** | **AIC (original data)** | **AIC (imputed data)** |
| --- | --- | --- | --- | --- |
| Influenza, Beh, subnational | 0.015 | 6.15 | 699.81 | 825.62 |
| Influenza, Beh, national | 0.020 | 5.85 | 610.37 | 727.19 |
| Influenza, Int, subnational | 0.04 | 4.72 | 1052.86 | 1290.27 |
| Influenza, Int, national | 0.06 | 4.93 | 990.43 | 1182.55 |
| Pneumoc., Beh, subnational | 0.08 | 5.61 | 231.44 | 455.19 |
| Pneumoc., Beh, national | 0.10 | 5.31 | 156.82 | 417.06 |

## *Short summary of results:*

The pattern of results for the 5C framework remained largely unaffected by data imputation, with results remaining stable, except for the 5C Calculation in the influenza uptake analysis from the subnational sample. Regarding the socio-demographic variables, some patterns shifted following data imputation. In the national sample, the number of doctor visits no longer showed a relationship with influenza vaccination behavior. In addition, education and job status no longer showed an association with influenza vaccination intention. For pneumococcal vaccination behavior, having children was no longer related to behavior. In the subnational sample, town size no longer correlated with influenza vaccination behavior. Furthermore, all levels of education demonstrated a relationship with the intention to receive the influenza vaccine, and a doctor's recommendation became a determinant of vaccination intention. Regarding pneumococcal vaccination behavior, marital status showed an association with behavior.

### Table S8

|  | **Flu Uptake Subnational, original data** | | | **Flu Uptake Subnational, imputed data** | | |
| --- | --- | --- | --- | --- | --- | --- |
| *Predictors* | *Odds Ratios* | *CI* | *p* | *Odds Ratios* | *CI* | *p* |
| Age | 1.05 | 1.02 – 1.08 | **0.001** | 1.05 | 1.02 – 1.08 | **0.001** |
| Gender (male vs. female) | 0.84 | 0.55 – 1.28 | 0.424 | 0.77 | 0.53 – 1.12 | 0.177 |
| Education = medium (vs. low) | 0.96 | 0.57 – 1.61 | 0.870 | 0.85 | 0.53 – 1.38 | 0.520 |
| Education = high (vs. low) | 1.40 | 0.88 – 2.21 | 0.153 | 1.19 | 0.78 – 1.80 | 0.423 |
| Jobstatus = working (vs. retired) | 0.70 | 0.38 – 1.27 | 0.238 | 0.64 | 0.36 – 1.13 | 0.124 |
| Marital status = single (vs. married) | 0.83 | 0.30 – 2.29 | 0.715 | 0.56 | 0.24 – 1.27 | 0.164 |
| Marital status = widowed (vs. married) | 1.08 | 0.39 – 3.00 | 0.878 | 0.62 | 0.28 – 1.36 | 0.235 |
| Insurance = statutory (vs. private) | 0.70 | 0.29 – 1.65 | 0.416 | 0.65 | 0.29 – 1.44 | 0.286 |
| Town size (small vs. large) | 1.50 | 1.01 – 2.24 | **0.047** | 1.24 | 0.87 – 1.79 | 0.238 |
| Living with partner (vs. living alone) | 0.53 | 0.19 – 1.42 | 0.205 | 0.93 | 0.43 – 2.01 | 0.855 |
| Having children (vs. not having children) | 0.79 | 0.35 – 1.74 | 0.551 | 0.86 | 0.42 – 1.77 | 0.690 |
| Physical health | 0.96 | 0.75 – 1.22 | 0.727 | 0.92 | 0.74 – 1.16 | 0.490 |
| No Chronic Diseases (vs. yes) | 1.09 | 0.70 – 1.69 | 0.693 | 1.16 | 0.78 – 1.73 | 0.458 |
| No recommendation to vaccinate (vs. rec by GP) | 1.49 | 0.81 – 2.76 | 0.206 | 1.48 | 0.86 – 2.59 | 0.160 |
| Doc. visit = Every 2-3 months (vs. <2-3 months) | 1.92 | 1.19 – 3.11 | **0.008** | 2.12 | 1.38 – 3.28 | **0.001** |
| Doc. visit = > 3 months (vs. <2-3 months) | 1.87 | 1.03 – 3.39 | **0.039** | 1.71 | 1.01 – 2.90 | **0.047** |
| Duration to reach doc = 6-10 min (vs. <5 min) | 1.19 | 0.71 – 1.97 | 0.508 | 1.01 | 0.64 – 1.60 | 0.962 |
| Duration to reach doc = >10 min (vs. <5 min) | 0.75 | 0.45 – 1.26 | 0.282 | 0.66 | 0.41 – 1.05 | 0.080 |
| 5C Coll.Responsibility | 1.42 | 1.14 – 1.78 | **0.002** | 1.30 | 1.07 – 1.60 | **0.011** |
| 5C Confidence | 1.83 | 1.57 – 2.17 | **<0.001** | 1.85 | 1.60 – 2.16 | **<0.001** |
| 5C Convenience | 0.85 | 0.62 – 1.15 | 0.297 | 0.88 | 0.67 – 1.15 | 0.353 |
| 5C Complacency | 0.94 | 0.82 – 1.08 | 0.360 | 0.90 | 0.79 – 1.02 | 0.088 |
| 5C Calculation | 0.89 | 0.79 – 1.00 | **0.045** | 0.91 | 0.82 – 1.02 | 0.103 |
| Observations | 590 | | | 698 | | |
| R2 Tjur | 0.250 | | | 0.241 | | |

### Table S9

|  | **Flu Intention Subnational, original data** | | | **Flu Intention Subnational, imputed data** | | |
| --- | --- | --- | --- | --- | --- | --- |
| *Predictors* | *Estimates* | *CI* | *p* | *Estimates* | *CI* | *p* |
| Age | 0.01 | -0.01 – 0.04 | 0.336 | 0.00 | -0.02 – 0.03 | 0.750 |
| Gender (male vs. female) | -0.25 | -0.67 – 0.18 | 0.254 | -0.33 | -0.70 – 0.04 | 0.081 |
| Education = medium (vs. low) | -0.48 | -0.98 – 0.02 | 0.061 | -0.50 | -0.95 – -0.05 | **0.029** |
| Education = high (vs. low) | -0.46 | -0.92 – 0.01 | 0.054 | -0.56 | -0.97 – -0.15 | **0.008** |
| Jobstatus = working (vs. retired) | -0.95 | -1.47 – -0.43 | **<0.001** | -0.84 | -1.31 – -0.37 | **<0.001** |
| Marital status = single (vs. married) | -0.37 | -1.25 – 0.50 | 0.402 | 0.03 | -0.64 – 0.70 | 0.926 |
| Marital status = widowed (vs. married) | -0.69 | -1.57 – 0.19 | 0.121 | -0.38 | -1.05 – 0.29 | 0.266 |
| Insurance = statutory (vs. private) | -0.04 | -0.80 – 0.71 | 0.910 | -0.34 | -1.01 – 0.33 | 0.317 |
| Town size (small vs. large) | 0.24 | -0.14 – 0.62 | 0.211 | 0.15 | -0.19 – 0.49 | 0.377 |
| Living with partner (vs. living alone) | 0.09 | -0.74 – 0.93 | 0.823 | 0.00 | -0.61 – 0.62 | 0.991 |
| Having children (vs. not having children) | 0.01 | -0.68 – 0.70 | 0.971 | 0.15 | -0.45 – 0.76 | 0.620 |
| Physical health | -0.16 | -0.41 – 0.08 | 0.196 | -0.14 | -0.36 – 0.09 | 0.229 |
| No Chronic Diseases (vs. yes) | 0.12 | -0.31 – 0.55 | 0.578 | 0.21 | -0.17 – 0.59 | 0.269 |
| No recommendation to vaccinate (vs. rec by GP) | 0.48 | -0.06 – 1.02 | 0.082 | 0.54 | 0.07 – 1.01 | **0.025** |
| Doc. visit = Every 2-3 months (vs. <2-3 months) | 0.27 | -0.17 – 0.72 | 0.230 | 0.31 | -0.08 – 0.70 | 0.121 |
| Doc. visit = > 3 months (vs. <2-3 months) | 0.19 | -0.37 – 0.75 | 0.503 | 0.03 | -0.46 – 0.52 | 0.893 |
| Duration to reach doc = 6-10 min (vs. <5 min) | 0.05 | -0.45 – 0.54 | 0.856 | 0.16 | -0.28 – 0.61 | 0.473 |
| Duration to reach doc = >10 min (vs. <5 min) | 0.06 | -0.43 – 0.55 | 0.806 | 0.08 | -0.36 – 0.52 | 0.718 |
| 5C Coll.Responsibility | 0.19 | 0.02 – 0.36 | **0.029** | 0.18 | 0.02 – 0.34 | **0.031** |
| 5C Confidence | 0.42 | 0.30 – 0.54 | **<0.001** | 0.43 | 0.32 – 0.55 | **<0.001** |
| 5C Convenience | -0.04 | -0.30 – 0.22 | 0.751 | -0.10 | -0.32 – 0.12 | 0.375 |
| 5C Complacency | -0.22 | -0.37 – -0.08 | **0.003** | -0.19 | -0.32 – -0.06 | **0.004** |
| 5C Calculation | 0.02 | -0.10 – 0.13 | 0.755 | 0.02 | -0.08 – 0.12 | 0.696 |
| Observations | 281 | | | 346 | | |
| R2 / R2 adjusted | 0.331 / 0.271 | | | 0.312 / 0.263 | | |

### Table S10

|  | **Flu Uptake National, original data** | | | **Flu Uptake National, imputed data** | | |
| --- | --- | --- | --- | --- | --- | --- |
| *Predictors* | *Odds Ratios* | *CI* | *p* | *Odds Ratios* | *CI* | *p* |
| Age | 1.02 | 0.98 – 1.05 | 0.309 | 1.02 | 0.99 – 1.05 | 0.130 |
| Gender (male vs. female) | 1.06 | 0.68 – 1.66 | 0.806 | 0.92 | 0.61 – 1.38 | 0.681 |
| Education = medium (vs. low) | 1.33 | 0.75 – 2.35 | 0.332 | 1.19 | 0.72 – 1.98 | 0.492 |
| Education = high (vs. low) | 1.11 | 0.64 – 1.90 | 0.717 | 0.99 | 0.60 – 1.63 | 0.970 |
| Jobstatus = working (vs. retired) | 1.23 | 0.62 – 2.44 | 0.553 | 0.90 | 0.49 – 1.65 | 0.733 |
| Marital status = single (vs. married) | 1.09 | 0.37 – 3.24 | 0.871 | 0.81 | 0.32 – 2.01 | 0.650 |
| Marital status = widowed (vs. married) | 2.42 | 0.77 – 7.88 | 0.136 | 1.54 | 0.59 – 4.06 | 0.377 |
| Insurance = statutory (vs. private) | 0.68 | 0.41 – 1.13 | 0.139 | 0.72 | 0.46 – 1.14 | 0.164 |
| Town size (small vs. large) | 1.73 | 1.11 – 2.70 | **0.016** | 1.57 | 1.06 – 2.34 | **0.026** |
| Living with partner (vs. living alone) | 0.54 | 0.18 – 1.56 | 0.261 | 0.75 | 0.31 – 1.81 | 0.527 |
| Having children (vs. not having children) | 1.40 | 0.74 – 2.67 | 0.302 | 1.31 | 0.74 – 2.34 | 0.357 |
| Physical health | 0.77 | 0.58 – 1.00 | 0.051 | 0.79 | 0.62 – 1.01 | 0.057 |
| No Chronic Diseases (vs. yes) | 1.65 | 1.05 – 2.61 | **0.030** | 1.57 | 1.04 – 2.38 | **0.032** |
| No recommendation to vaccinate (vs. rec by GP= | 2.11 | 1.25 – 3.61 | **0.006** | 1.92 | 1.20 – 3.09 | **0.007** |
| Doc. visit = Every 2-3 months (vs. <2-3 months) | 1.64 | 1.01 – 2.67 | **0.043** | 1.52 | 0.98 – 2.36 | 0.061 |
| Doc. visit = > 3 months (vs. <2-3 months) | 1.39 | 0.72 – 2.72 | 0.335 | 1.18 | 0.66 – 2.14 | 0.578 |
| Duration to reach doc = 6-10 min (vs. <5 min) | 1.00 | 0.59 – 1.71 | 0.992 | 1.01 | 0.63 – 1.63 | 0.966 |
| Duration to reach doc = >10 min (vs. <5 min) | 1.23 | 0.71 – 2.12 | 0.456 | 1.23 | 0.75 – 2.02 | 0.404 |
| 5C Coll. Responsibility | 1.00 | 0.80 – 1.25 | 0.989 | 0.91 | 0.75 – 1.10 | 0.330 |
| 5C Confidence | 2.06 | 1.77 – 2.43 | **<0.001** | 2.04 | 1.77 – 2.38 | **<0.001** |
| 5C Convenience | 0.64 | 0.44 – 0.89 | **0.012** | 0.67 | 0.47 – 0.91 | **0.015** |
| 5C Complacency | 0.75 | 0.63 – 0.88 | **0.001** | 0.73 | 0.62 – 0.85 | **<0.001** |
| 5C Calculation | 0.72 | 0.62 – 0.82 | **<0.001** | 0.75 | 0.67 – 0.85 | **<0.001** |
| Observations | 600 | | | 701 | | |
| R2 Tjur | 0.389 | | | 0.364 | | |

### Table S11

|  | **Flu Intention National**  **original data** | | | **Flu Intention National**  **imputed data** | | |
| --- | --- | --- | --- | --- | --- | --- |
| *Predictors* | *Estimates* | *CI* | *p* | *Estimates* | *CI* | *p* |
| Age | -0.00 | -0.02 – 0.02 | 0.916 | -0.01 | -0.03 – 0.01 | 0.444 |
| Gender (male vs. female) | 0.08 | -0.23 – 0.39 | 0.616 | -0.05 | -0.33 – 0.23 | 0.742 |
| Education = medium (vs. low) | -0.41 | -0.80 – -0.02 | **0.039** | -0.18 | -0.53 – 0.16 | 0.291 |
| Education = high (vs. low) | -0.22 | -0.62 – 0.19 | 0.290 | -0.16 | -0.52 – 0.21 | 0.398 |
| Jobstatus = working (vs. retired) | 0.47 | 0.07 – 0.87 | **0.022** | 0.22 | -0.14 – 0.57 | 0.238 |
| Marital status = single (vs. married) | 0.03 | -0.66 – 0.72 | 0.934 | 0.06 | -0.51 – 0.62 | 0.848 |
| Marital status = widowed (vs. married) | 0.20 | -0.54 – 0.94 | 0.596 | 0.06 | -0.54 – 0.66 | 0.841 |
| Insurance = statutory (vs. private) | -0.05 | -0.40 – 0.29 | 0.765 | -0.01 | -0.32 – 0.30 | 0.953 |
| Town size (small vs. large) | 0.25 | -0.06 – 0.56 | 0.115 | 0.16 | -0.11 – 0.44 | 0.242 |
| Living with partner (vs. living alone) | -0.37 | -1.04 – 0.30 | 0.279 | -0.25 | -0.79 – 0.29 | 0.360 |
| Having children (vs. not having children) | 0.04 | -0.39 – 0.47 | 0.858 | 0.17 | -0.22 – 0.55 | 0.387 |
| Physical health | 0.04 | -0.15 – 0.22 | 0.710 | 0.03 | -0.13 – 0.20 | 0.690 |
| No Chronic Diseases (vs. yes) | -0.03 | -0.34 – 0.29 | 0.870 | 0.02 | -0.26 – 0.31 | 0.878 |
| No recommendation to vaccinate (vs. rec by GP) | 0.36 | 0.04 – 0.69 | **0.027** | 0.34 | 0.05 – 0.62 | **0.023** |
| Doc. visit = Every 2-3 months (vs. <2-3 months) | 0.04 | -0.30 – 0.38 | 0.825 | 0.09 | -0.21 – 0.40 | 0.555 |
| Doc. visit = > 3 months (vs. <2-3 months) | 0.29 | -0.22 – 0.79 | 0.266 | 0.14 | -0.30 – 0.57 | 0.528 |
| Duration to reach doc = 6-10 min (vs. <5 min) | 0.17 | -0.20 – 0.53 | 0.374 | 0.16 | -0.17 – 0.49 | 0.336 |
| Duration to reach doc = >10 min (vs. <5 min) | 0.03 | -0.34 – 0.40 | 0.869 | 0.07 | -0.27 – 0.41 | 0.703 |
| 5C Coll.Responsibility | 0.09 | -0.05 – 0.22 | 0.200 | 0.06 | -0.07 – 0.18 | 0.362 |
| 5C Confidence | 0.34 | 0.24 – 0.43 | **<0.001** | 0.29 | 0.20 – 0.38 | **<0.001** |
| 5C Convenience | -0.10 | -0.30 – 0.10 | 0.336 | -0.11 | -0.30 – 0.07 | 0.222 |
| 5C Complacency | -0.41 | -0.55 – -0.26 | **<0.001** | -0.41 | -0.55 – -0.28 | **<0.001** |
| 5C Calculation | -0.11 | -0.22 – -0.01 | **0.031** | -0.09 | -0.19 – -0.00 | **0.049** |
| Observations | 298 | | | 359 | | |
| R2 / R2 adjusted | 0.336 / 0.280 | | | 0.279 / 0.229 | | |

### Table S12

|  | **Pneumo Uptake Subnational**  **original data** | | | **Pneumo Uptake Subnational**  **imputed data** | | |
| --- | --- | --- | --- | --- | --- | --- |
| *Predictors* | *Odds Ratios* | *CI* | *p* | *Odds Ratios* | *CI* | *p* |
| Age | 1.03 | 0.97 – 1.09 | 0.390 | 1.02 | 0.98 – 1.06 | 0.328 |
| Gender (male vs. female) | 1.09 | 0.45 – 2.64 | 0.850 | 1.11 | 0.62 – 1.98 | 0.728 |
| Education = medium (vs. low) | 0.97 | 0.34 – 2.77 | 0.947 | 1.38 | 0.66 – 2.88 | 0.394 |
| Education = high (vs. low) | 0.91 | 0.36 – 2.29 | 0.834 | 1.38 | 0.76 – 2.54 | 0.293 |
| Jobstatus = working (vs. retired) | 0.58 | 0.17 – 1.97 | 0.381 | 0.51 | 0.19 – 1.26 | 0.154 |
| Marital status = single (vs. married) | 0.27 | 0.03 – 1.96 | 0.203 | 0.31 | 0.10 – 0.97 | **0.048** |
| Marital status = widowed (vs. married) | 0.29 | 0.04 – 2.23 | 0.229 | 0.46 | 0.14 – 1.46 | 0.190 |
| Insurance = statutory (vs. private) | 0.81 | 0.14 – 5.44 | 0.822 | 0.70 | 0.19 – 2.44 | 0.585 |
| Town size (small vs. large) | 3.48 | 1.47 – 8.73 | **0.006** | 2.24 | 1.28 – 3.98 | **0.005** |
| Living with partner (vs. living alone) | 4.20 | 0.63 – 30.05 | 0.137 | 2.22 | 0.73 – 6.80 | 0.161 |
| Having children (vs. not having children) | 2.54 | 0.32 – 36.61 | 0.427 | 2.01 | 0.72 – 5.50 | 0.179 |
| Physical health | 0.83 | 0.48 – 1.42 | 0.496 | 1.14 | 0.80 – 1.63 | 0.461 |
| No Chronic Diseases (vs. yes) | 0.39 | 0.15 – 0.94 | **0.041** | 0.63 | 0.34 – 1.13 | 0.122 |
| No recommendation to vaccinate (vs. rec by GP) | 13.52 | 6.05 – 32.87 | **<0.001** | 40.85 | 23.77 – 73.59 | **<0.001** |
| Doc. visit = Every 2-3 months (vs. <2-3 months) | 1.50 | 0.57 – 3.96 | 0.410 | 2.03 | 1.10 – 3.81 | **0.025** |
| Doc. visit = > 3 months (vs. <2-3 months) | 1.02 | 0.30 – 3.42 | 0.976 | 1.48 | 0.68 – 3.27 | 0.327 |
| Duration to reach doc = 6-10 min (vs. <5 min) | 0.88 | 0.30 – 2.51 | 0.805 | 0.78 | 0.39 – 1.52 | 0.459 |
| Duration to reach doc = >10 min (vs. <5 min) | 0.62 | 0.20 – 1.85 | 0.394 | 0.95 | 0.48 – 1.88 | 0.882 |
| 5C Coll.Responsibility | 1.32 | 0.80 – 2.14 | 0.268 | 1.15 | 0.77 – 1.70 | 0.488 |
| 5C Confidence | 1.96 | 1.35 – 2.96 | **0.001** | 2.00 | 1.42 – 2.88 | **<0.001** |
| 5C Convenience | 0.75 | 0.45 – 1.22 | 0.266 | 0.86 | 0.57 – 1.31 | 0.488 |
| 5C Complacency | 0.71 | 0.54 – 0.92 | **0.011** | 0.70 | 0.55 – 0.88 | **0.003** |
| 5C Calculation | 0.81 | 0.53 – 1.21 | 0.314 | 0.85 | 0.58 – 1.20 | 0.364 |
| Observations | 218 | | | 620 | | |
| R2 Tjur | 0.451 | | | 0.531 | | |

### Table S13

|  | **Pneumo Uptake National**  **original data** | | | **Pneumo Uptake National**  **imputed data** | | |
| --- | --- | --- | --- | --- | --- | --- |
| *Predictors* | *Odds Ratios* | *CI* | *p* | *Odds Ratios* | *CI* | *p* |
| Age | 1.13 | 1.04 – 1.23 | **0.006** | 1.07 | 1.02 – 1.11 | **0.002** |
| Gender (male vs. female) | 0.74 | 0.19 – 2.63 | 0.641 | 1.08 | 0.60 – 1.95 | 0.806 |
| Education = medium (vs. low) | 1.51 | 0.35 – 6.90 | 0.582 | 0.63 | 0.29 – 1.33 | 0.233 |
| Education = high (vs. low) | 0.94 | 0.21 – 4.07 | 0.936 | 1.11 | 0.53 – 2.29 | 0.776 |
| Jobstatus = working (vs. retired) | 2.10 | 0.32 – 15.41 | 0.447 | 0.86 | 0.31 – 2.26 | 0.772 |
| Marital status = single (vs. married) | 1.59 | 0.11 – 32.70 | 0.750 | 1.04 | 0.25 – 3.97 | 0.955 |
| Marital status = widowed (vs. married) | 3.66 | 0.31 – 65.81 | 0.339 | 1.24 | 0.32 – 4.66 | 0.751 |
| Insurance = statutory (vs. private) | 1.32 | 0.33 – 5.48 | 0.698 | 1.26 | 0.64 – 2.46 | 0.505 |
| Town size (small vs. large) | 0.64 | 0.19 – 2.05 | 0.464 | 0.95 | 0.53 – 1.73 | 0.869 |
| Living with partner (vs. living alone) | 0.40 | 0.03 – 3.84 | 0.450 | 0.58 | 0.17 – 2.08 | 0.403 |
| Having children (vs. not having children) | 5.87 | 1.12 – 36.62 | **0.045** | 1.75 | 0.79 – 3.87 | 0.168 |
| Physical health | 1.07 | 0.49 – 2.38 | 0.862 | 0.71 | 0.49 – 1.02 | 0.070 |
| No Chronic Diseases (vs. yes) | 2.03 | 0.60 – 7.38 | 0.264 | 1.43 | 0.78 – 2.63 | 0.251 |
| No recommendation to vaccinate (vs. rec by GP) | 15.90 | 5.27 – 57.44 | **<0.001** | 33.76 | 19.41 – 61.23 | **<0.001** |
| Doc. visit = Every 2-3 months (vs. <2-3 months) | 2.31 | 0.66 – 8.52 | 0.193 | 1.25 | 0.65 – 2.43 | 0.502 |
| Doc. visit = > 3 months (vs. <2-3 months) | 2.10 | 0.35 – 15.03 | 0.433 | 1.43 | 0.59 – 3.42 | 0.422 |
| Duration to reach doc = 6-10 min (vs. <5 min) | 0.74 | 0.17 – 3.19 | 0.689 | 1.24 | 0.61 – 2.56 | 0.559 |
| Duration to reach doc = >10 min (vs. <5 min) | 0.98 | 0.22 – 4.38 | 0.982 | 1.07 | 0.52 – 2.22 | 0.855 |
| 5C Coll.Responsibility | 1.48 | 0.81 – 2.83 | 0.214 | 1.28 | 0.73 – 2.40 | 0.412 |
| 5C Confidence | 1.31 | 0.81 – 2.18 | 0.289 | 1.39 | 0.91 – 2.14 | 0.132 |
| 5C Convenience | 0.69 | 0.40 – 1.18 | 0.176 | 0.78 | 0.47 – 1.29 | 0.320 |
| 5C Complacency | 0.65 | 0.46 – 0.90 | **0.011** | 0.61 | 0.45 – 0.82 | **0.001** |
| 5C Calculation | 1.01 | 0.66 – 1.52 | 0.950 | 0.88 | 0.61 – 1.27 | 0.503 |
| Observations | 144 | | | 650 | | |
| R2 Tjur | 0.515 | | | 0.504 | | |
